# A naturally hypersensitive porcine model may help understand the mechanism of COVID-19 mRNA vaccine-induced rare (pseudo) allergic reactions: complement activation as a possible contributing factor

**DOI:** 10.1007/s11357-021-00495-y

**Published:** 2022-02-11

**Authors:** László Dézsi, Tamás Mészáros, Gergely Kozma, Mária H-Velkei, Csaba Zs. Oláh, Miklós Szabó, Zsófia Patkó, Tamás Fülöp, Mark Hennies, Miklós Szebeni, Bálint András Barta, Béla Merkely, Tamás Radovits, János Szebeni

**Affiliations:** 1 Nanomedicine Research and Education Center, Department of Translational Medicine, Semmelweis University, Budapest, Hungary; 2 SeroScience LCC, Budapest, Hungary; 3 Department of Neurosurgery, BAZ County Central Hospital and Borsod County University Teaching Hospital, Miskolc, Hungary; 4 Department of Pulmonology, BAZ County Central Hospital and Borsod County University Teaching Hospital, Miskolc, Hungary; 5 Department of Radiology, BAZ County Central Hospital and Borsod County University Teaching Hospital, Miskolc, Hungary; 6 TECOdevelopment GmbH, Rheinbach, Germany; 7 Heart and Vascular Center, Semmelweis University, Budapest, Hungary; 8 Department of Nanobiotechnology and Regenerative Medicine, Faculty of Health, Miskolc University, Miskolc, Hungary

**Keywords:** COVID-19, CARPA, Complement, Anaphylatoxins, Pseudoallergy, Shock, Hemodynamic changes, Pigs

## Abstract

A tiny fraction of people immunized with lipid nanoparticle (LNP)-enclosed mRNA (LNP-mRNA) vaccines develop allergic symptoms following their first or subsequent vaccinations, including anaphylaxis. These reactions resemble complement (C) activation-related pseudoallergy (CARPA) to i.v. administered liposomes, for which pigs provide a naturally oversensitive model. Using this model, we injected i.v. the human vaccination dose (HVD) of BNT162b2 (Comirnaty, CMT) or its 2-fold (2x) or 5-fold (5x) amounts and measured the hemodynamic changes and other parameters of CARPA. We observed in 6 of 14 pigs transient pulmonary hypertension along with thromboxane A2 release into the blood and other hemodynamic and blood cell changes, including hypertension, granulocytosis, lymphopenia, and thrombocytopenia. One pig injected with 5x CMT developed an anaphylactic shock requiring resuscitation, while a repeat dose failed to induce the reaction, implying tachyphylaxis. These typical CARPA symptoms could not be linked to animal age, sex, prior immune stimulation with zymosan, immunization of animals with Comirnaty i.v., or i.m. 2 weeks before the vaccine challenge, and anti-PEG IgM levels in Comirnaty-immunized pigs. Nevertheless, IgM binding to the whole vaccine, used as antigen in an ELISA, was significantly higher in reactive animals compared to non-reactive ones. Incubation of Comirnaty with pig serum in vitro showed significant elevations of C3a anaphylatoxin and sC5b-9, the C-terminal complex. These data raise the possibility that C activation plays a causal or contributing role in the rare HSRs to Comirnaty and other vaccines with similar side effects. Further studies are needed to uncover the factors controlling these vaccine reactions in pigs and to understand their translational value to humans.

## Introduction

Beyond efficacy, a major contributor to the success of COVID-19 vaccines, including Pfizer/BioNTech’s BNT162b2, an mRNA-lipid nanoparticle (mRNA-LNP)-based vaccine also called Comirnaty (CMT), is their safety in the overwhelming majority of vaccinated people [1]. However, a tiny fraction of vaccine recipients develop local and/or systemic allergy, also known as hypersensitivity reaction (HSR). These reactions arise within minutes to hours after vaccination, and in most cases, they are controllable and fade without the need for intervention. However, occasionally, they can be severe or even escalate into anaphylaxis with shock, requiring emergency measures beyond epinephrin injection [2–11]. Although all COVID-19 vaccines can cause severe adverse effects (SAEs) in an occasional patient, and LNP-mRNA vaccines have actually more favorable statistics in this regard than the vector vaccines [12], the present study focused on the HSRs to Comirnaty, as it consists of PEGylated LNPs that resemble PEGylated liposomes, and “lessons” learned from nanomedicine research suggest that such nanoparticles (NPs) injected into the blood can cause so-called “infusion reactions” whose symptoms are very similar, or the same as those reported for the HSRs to LNP-mRNA vaccines [13, 14]. These symptoms include, but not limited, to sudden tachycardia/palpitation, dyspnea/tachypnea/apnea, hypo/hypertension, generalized or local flushing or rash, face/throat/tongue/lip swelling (i.e., angioedema), chest/back/abdominal pain/tightness, light-headedness/panic [2–11]. They can be explained, at least in part, with complement (C) activation-related anaphylatoxin (C3a, C4a, and C5a) release, but not with IgE-mediated type-I allergy, leading to the term, C activation-related pseudoallergy (CARPA) [13, 15]. An important lesson learned from nanomedicine research is that CARPA can be uniquely modeled in pigs because this species has an inborn hypersensitivity to i.v. administered liposomes and other NPs [15–18]. This resemblance of symptoms provided a rationale to use the porcine CARPA model to understand the mechanism of LNP-mRNA vaccine-induced HSRs.

As the first approach, we administered the human vaccine dose (HVD) and its multiples in i.v. bolus to maximize the chance to see CARPA with the clear understanding that this treatment does not exactly imitate the human practice of injecting the HVD in the deltoid muscle i.m. It was contemplated that if we could provoke the reactions under the above conditions, it would be the next challenge to refine the model to better mimic the human administration conditions. Nevertheless, it was also clear that if we saw HSRs at all, that would prove our capability to reproduce a very rare adverse reaction of a vaccine in an animal model. In addition to patient analysis, studies in animal models are essential to understand these reactions, but in vivo modeling of rare toxicities is inherently difficult because of the large number of animals needed to mimic the low prevalence of adverse symptoms. Thus, the use of the sensitized or naturally sensitive animal models, such as the pig in the present study, may be a prerequisite for achieving such goals.

As detailed below, the experiments did show more or less expressed HSR symptoms in 6 of 14 pigs, with one animal undergoing anaphylactic shock requiring resuscitation. Together with the strong C activation by Comirnaty in pig serum in vitro, these data point to the possible involvement of CARPA in the anaphylaxis and other allergic symptoms caused by this vaccine. Thus, the initial steps have been taken to establish an animal model that may help solve the vaccine reaction problem.

## Materials and methods

### Materials

Comirnaty was from Pfizer/BioNTech, the vaccine used for human vaccinations against SARS-Cov-2 infections. One 0.3 mL shot contains, in addition to phosphate buffer and sucrose, 30 μg mRNA, 430 μg ALC-0315, (4-hydroxybutyl) azanediyl)bis (hexane-6,1-diyl)bis(2-hexyldecanoate); 50 μg ALC-0159, 2-[(polyethylene glycol)-2000]-N,N ditetradecylacetamide 90; μg 1,2-Distearoyl-sn-glycero-3-phosphocholine (DSPC); and 200 μg cholesterol. Its total lipid content is 0.77 mg. The porcine C3a kit was obtained from TECO*Medical* AG, Sissach, Switzerland (Cat No: TE1078). Commercial Doxil (Caelyx) was obtained from the pharmacy of Semmelweis University. Zymosan, Dulbecco’s phosphate-buffered saline (PBS) without Ca^2+^/Mg^2+^ and bovine calf serum, and biotin-labeled goat polyclonal anti-porcine IgM were from Sigma Chemical Co. (St. Louis, MO, USA).

### Methods

#### Animals and groups

Landrace pigs were obtained from the Research Institute for Animal Breeding, Nutrition, and Meat Science of the Hungarian University of Agriculture and Life Sciences (Herceghalom, Hungary). The study involved 8 female and 6 castrated male pigs in the 22–65 kg size range, which were selected into four groups: 1) naïve, 2) zymosan pretreated (innate immune pre-stimulated), 3) i.v. treated (“immunized”) with Comirnaty, and 4) i.m. immunized with Comirnaty. Since the experiments were intended as a pilot exploration of Comirnaty-induced allergy symptoms using cardiovascular and other endpoints, sex, or body weights (age) were not criteria for preselection into these groups.

The investigation conformed to the EU Directive 2010/63/EU and the Guide for the Care and Use of Laboratory Animals used by the US National Institutes of Health (NIH Publication no. 85–23, revised 1996). The experiments were approved by the Ethical Committee of Hungary for Animal Experimentation (permission numbers: PE/EA/1177-4/2021 and BORS-02/2021).

#### The porcine CARPA model

The model was described in several studies earlier [15–18]. In brief, pigs were sedated with i.m. ketamine/xylazine and then anesthetized with isoflurane (2 − 3% in O_2_). Intubation was performed with endotracheal tubes to maintain free airways and to enable controlled ventilation if necessary. The animals were breathing spontaneously during the experiments. Surgery was done after povidone-iodine (10%) disinfection of the skin. In order to measure the pulmonary arterial pressure (PAP), a Swan-Ganz catheter (AI-07124, 5 Fr. 110 cm, Arrow Internat Inc.) was introduced into the pulmonary artery via the right external jugular vein. Additional catheters were placed into the left femoral artery to record the systemic arterial pressure (SAP), into the left external jugular vein for saline and drug administration, and into the left femoral vein for blood sampling. Hemodynamic and ECG data were collected using instruments from Pulsion Medical Systems and Powerlab, AD-Instruments (Castle Hill, Australia). At the end of the experiments, animals were sacrificed with Euthasol and concentrated potassium chloride.

#### I.v. challenge with Comirnaty and zymosan

Animals were injected into the left external jugular with 2 sequential i.v. boluses of Comirnaty. The storage, thawing, and dilution procedures corresponded to the human application [19], except that the vaccine was flushed into the circulation with 5 mL saline. The dose was the full HVD or two or five-fold this amount, referred to as 1x , 2x , and 5x HVD. At the end of the experiment, the animals were injected with 0.1 mg/kg zymosan to provide a positive control for CARPA. The initial 1x HVD was chosen as the possible maximal dose reaching the blood of a young man in case of accidental injection of the full dose into a blood vessel in the deltoid muscle, e.g., collateral branches of the circumflex humeral arteries or veins, possibly the deltoid and/or acromial arteries or veins.

#### Scoring of cardiopulmonary distress

The intensity of CARPA was quantified by the cardiac abnormality score (CAS), established earlier for liposome-induced cardiopulmonary distress in pigs [20]. It is an arbitrary rank between 1 and 5, specifying minimal, mild, moderate, severe, and lethal reactions, respectively. These categories were based on a combination of all measurable manifestations (SAP, PAP, HR, and ECG) of cardiopulmonary distress during porcine CARPA [20].

#### Innate immune preconditioning with zymosan

The pigs in this group were injected with 1 mg/kg zymosan 22–49 days before these studies, as an independent experiment with the goal of inducing cytokine storm. These animals were subjected to blood withdrawals every 3 days for 15 days after the zymosan treatment to measure innate immune activation (complement and cytokine levels). These data will be presented elsewhere; in the present experiment, these pigs served to provide a model for innate immune stimulation before challenging with Comirnaty.

#### Immunization with Comirnaty

The immunization with the HVD of Comirnaty was done either i.v. or i.m., by injections into the ear vein or the supraspinatus muscle of the animals, respectively.

#### Measurement of anti-PEG and anti-Comirnaty IgM in pig blood

Serial measurements of blood anti-PEG IgM levels after immunization were performed using an ELISA, as described earlier [21]. In short, Polysorp (Nunc) plates were coated with 2.0 μg/well DSPE-PEG2000 in 100 μL of bicarbonate buffer (7.14 μM) (pH∼9.0) overnight at 4 °C, followed by blocking of the wells with 150 μL of PBS/0.05% Tween-20 + 2% bovine serum albumin (BSA) at 37 °C for 1 h. Before blocking, wells were washed 3 × with 300 μL of wash buffer containing PBS/0.05% Tween-20 for 1 min. The EDTA-anti-coagulated plasma samples were diluted by PBS/0.05% Tween-20 + 1% BSA in the 20–3000-fold range and incubated in the wells for 1.5 h at 37 °C, with slow shaking. Wells were washed 5 × with 300 μL of wash buffer for 1 min. After staining with 100 μL of HRP-conjugated anti-porcine IgM (15,000 x dilution, Sigma) or IgG (800 × dilution, Sigma) for 1 h, wells were washed again 5 × with wash buffer as mentioned. The antibodies were stained by incubation with 100 μL of substrate solution (Neogen) containing 3,3′,5,5′-tetramethylbenzidine (TMB) and hydrogen peroxide for 15 min in dark. The reaction was stopped with 50 μL of 2NH_2_SO_4_ and A_450_ was read with a FLUOstar Omega 96-well plate reader (BMG Labtech, Ortenberg, Germany). One tenth of titer untit was defined as the dilution at which the blank-corrected OD of a dedicated reference pig plasma, used as a standard of anti-PEG IgM, was equal to the 10-fold of blank. The specified anti-PEG values represent readings in the linear section of the calibration curve.

A similar ELISA was used to measure the levels of all IgM (not only PEG) binding to the vaccine before the i.v. challenge with Comirnaty (anti-CMT IgM), except that the plates were coated with Comirnaty as antigen.

#### Measurement of complement activation by Comirnaty in pig serum in vitro

Freshly drawn blood from healthy pigs was let to clot at room temperature (RT), the serum was separated by centrifugation, aliquoted, and then stored at − 70 °C until the C activation studies. The latter included incubation with Comirnaty (1:1.5 volume ratio) and control zymosan (0.1 mg/mL) for the specified time at 37 °C with shaking. The reaction was stopped by diluting the samples by stop solution containing 0.05% Tween-20 and 20 mM EDTA. The liberated C3a and sC5b-9, i.e., biomarkers of C activation, were determined as follows. Porcine C3a was measured using a capture EIA (TECO *Medical* AG, Sissach, Switzerland, ref: TE1078) following the manufacturer’s instructions. Briefly, the serum samples diluted by the stop solution were incubated in the wells (100 µl/well) in plates coated with 50 ng/ml Ab2 (a porcine C3a specific antibody) for 2 h at RT, followed by washings 5 × with washing buffer. This was followed by incubation with biotinylated Ab1 (100 µl/well, 25 ng/ml), for 2 h at 4 °C while shaking and 3 × washings again. Next, streptavidin horseradish peroxidase conjugate (100 ng/ml, 100 µl/well) was added and incubated for 2 h RT while shaking. In the final step, after a 3 × wash, 100 µl TMB was added and incubated for 30 min on RT on a shaker. The reaction was stopped with HCL solution and the plate was read using FLUOstar Omega 96-well plate reader (BMG Labtech, Ortenberg, Germany) at 450 nm. The C3a concentrations were given in arbitrary units (AU/ml).

Porcine sC5b-9 was measured as described earlier [21, 22]. In brief, microtiter plates were coated with mouse anti-human sC5b-9 ascites (clone aE11) and incubated for 1 h with pig plasma containing 10 mM EDTA. The second Ab, biotinylated mouse anti-human C6 (Quidel A219), was stained with streptavidin − horseradish peroxidase using ABTS and H_2_O_2_ substrate.

#### Statistical methods

To get updated prevalence data on vaccine-induced anaphylaxis, we used the Vaccine Adverse Event Reporting System (VAERS), a data bank for all adverse effects of US-licensed vaccines [23–25]. The different synonyms of the same reaction were captured with an algorithm formulated in PostgreSQL [26] to include all terms related to anaphylaxis (e.g., anaphylactic or anaphylactoid) and/or shock (all vaccine-induced shocks are anaphylactic by nature, assuming no major simultaneous loss of blood). Redundant symptoms or symptom synonyms were then excluded from the reaction count. The analysis involved downloading of 3 VAERS files for each year in question (e.g., “2021VAERSDATA,” “2021VAERSVAX,” and “2021VAERSSYMTOMS”), aggregating data across all years, grouping by unique VAERS_ID (i.e., grouping per adverse event), and querying for the terms of interest.

Differences among all other analytes in this study were analyzed by ANOVA followed by post hoc tests, or other tests, as specified in the figure legends. A *p*-value of < 0.05 was considered statistically significant. Statistical analyses were performed by GraphPad Prism software (GraphPad Software, La Jolla, CA, USA).

## Results

### Pathophysiological changes caused by i.v. administration of Comirnaty in pigs

#### Illustration of Comirnaty-induced acute hemodynamic changes in 3 pigs as a function of dose

**Fig. 1:**
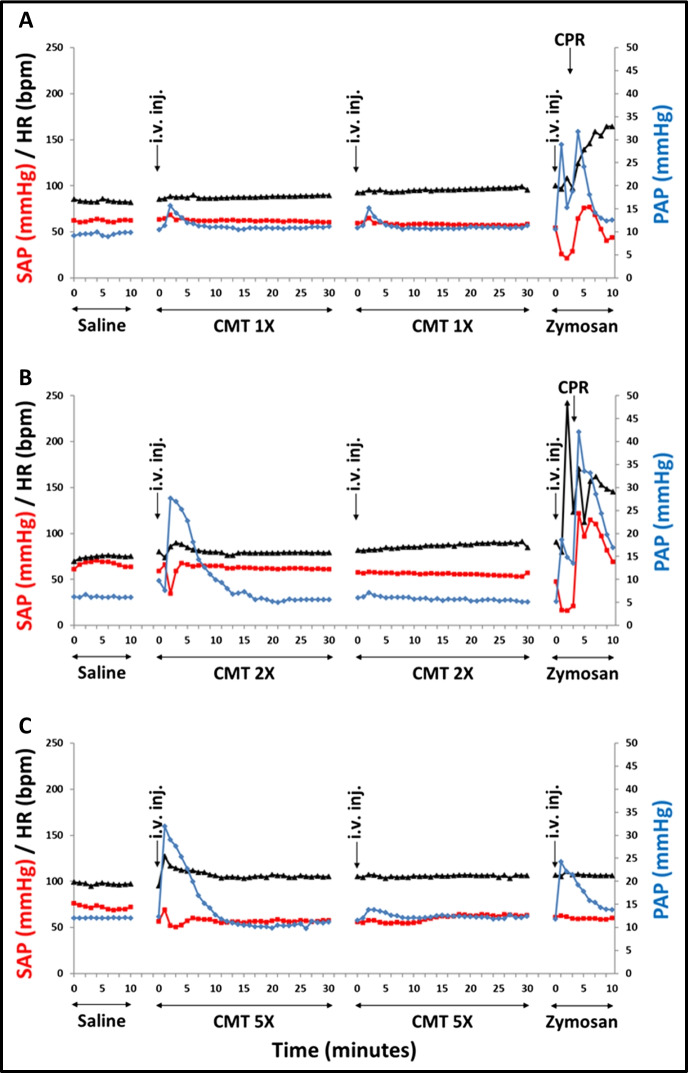
Time courses of mean systemic (SAP) and pulmonary (PAP) arterial blood pressure and heart rate (HR) changes following 2 consecutive i.v. injections of pigs with 1x (Panel **A**), 2x (**B**), and 5x (**C**) human vaccine dose of Comirnaty. The timing of injections and length of observations are shown by arrows. The y-axis gives the units, separately for SAP and HR (left y-axis) and PAP (right y-axis). Panels **A** and **B** show animals pretreated with zymosan 46 and 49 days earlier (pig no. 1 and 2, respectively). Panel **C** was a naïve pig (no. 10). All animals were injected with 0.1 mg/kg zymosan at the end of the experiments. The HVD expressed as mg/kg for each vaccine component is specified in Table 2. Abbreviations: *i.v. inj*, intravenous injection

Figure 1A–C shows the PAP, SAP, and HR changes caused by i.v. injection of 1x  , 2x , and 5x HVD of Comirnaty, respectively. The HVD (1x HVD, Panel A) led to a small, but the clearly distinguishable rise of PAP (from 10 to 15 mmHg) in the 2–5 min. time window, 2x  HVD (B) triggered a major rise of PAP with paralleling minor drop of SAP, and 5x HVD (C) caused similar reaction as 2x HVD, although the reaction to zymosan was less expressed. The second repeat dose of Comirnaty reproduced the first reaction in A, but it caused no reaction in B and C, indicating self-induced tolerance, known as tachyphylaxis. The fact that the 1x Comirnaty-caused minimal PAP rise did not disappear after the second injection suggests that tachyphylaxis is a dose dependent process. The third, zymosan positive control was comparable for animals A and B, but visibly smaller in C, which could be explained with intrinsically weaker sensitivity to CARPA and/or extension of the first dose’s tolerogenic effect to zymosan.

The physical parameters (body weight and sex) of the above 3 and additional 11 pigs used in this study are shown in Table 1, together with quantitation of cardiopulmonary, skin, immunological, hemodynamic, and hematological endpoints before and after Comirnaty challenges.

**Table 1:** Animal specifics, treatment parameters, HSR grades, presence of skin reaction, hemodynamic and blood cell changes caused by i.v. bolus of Comirnaty in pigs

	Animal specs				Treatment parameters				HSR CAS			AVA IgM		Blood pressure		Blood cells					
Exp #	Pig no	kg	Sex	Zym (mg/kg)	Imm. w CMT	Postzym days	Imm. days	CMT x HVD	1st inj	2nd inj	Skin flush	Anti-PEG	Anti-CMT	PAP	SAP	Granulocyte		Lymphocyte		Thrombocyte	
														↑	↑	Min	Max	Min	Max	Min	Max
1	10	22.4	f	0	None			5	3	0		471.0	0.2	*260*	100	− 17.8	46.4	− 18.6	− 1.0	− 9.8	× − 3.7
2	11	23.8	f	0	None			5	0	0		208.0	0.1	106	100	− 27.1	43.6	− 15.4	2.0	− 9.4	− 6.8
3	12	25.4	m	0	None			5	0	0		ND	0.1	110	100	− 0.1	85.7	− 11.9	1.5	− 8.9	7.3
4	1	65.0		1	None	46		1	1	1		ND	0.4	*150*	100	− 9.8	− 2.3	− 21.0	− 14.4	− 7.1	4.1
5	2	56.0		1	None	49		2	2	0	X	ND	1.0	*300*	100	0.2	20.8	− 27.4	− 9.7	4.7	3.7
6	13	39.8	m	1	None	22		1	0	0		2.6	0.3	110	100	− 16.8	16.2	− 26.4	− 3.9	− 8.4	7.2
7	14	35.2	m	1	None	23		3	2	0		1.6	0.2	120	121	− 28.9	43.4	− 27.2	− 22.4	− 11.8	7.9
8	15	38.4	1	1	None	24		10	0	0		606.0	1.6	107	100	− 6.8	− 0.9	− 16.0	− 9.2	− 14.8	0.0
9	3	27.0	m	0	I.v.		18	5	0	0		589.0	0.1	100	100	− 11.8	72.8	− 1.7	16.1	3.1	8.3
10	4	29.8	m	0	I.v.		19	5	0	0		147.0	0.1	100	100	− 8.4	5.8	− 30.0	− 13.6	− 22.3	− 3.4
11 ANA	*9*	28.8	*m*	0	I.v.		14	5	5	0	X	*237.0*	*0.2*	*250*	100	*− 14.3*	*48.7*	*− 17.9*	*23.1*	*− 20.8*	*− 1.0*
12	6	29.8	1	0	I.m.		20	5	1	0		666.0	0.1	120	100	16.8	65.7	− 8.9	6.0	− 4.2	5.0
13	7	33.2	1	0	I.m.		14	5	0	0		785.0	0.2	110	100	− 14.0	− 0.2	− 19.2	− 3.1	− 18.7	2.4
14	8	26.4	*1*	0	I.m.		14	5	0	0		563.0	0.1	110	100	− 12.0	− 4.9	− 8.7	− 3.6	− 3.5	3.0

The experiment (Exp) number (#) is shown for reference, the actual sequence of experiments is specified by the pig numbers (Pig no). Italicized values show ≥ 20% changes consistently in one direction (i.e. rise or fall).  These were arbitrarily considered as signs of immune reactivity, although they are biologically not necessarily significant or clinically manifest. Blood pressure and blood cell entries are expressed as % relative to baseline, negative values mean decrease. Min and max imply minimal and maximal readings. Abbreviations: *zym*, zymosan; *CMT*, Comirnaty; *post zym days*, days between experiment and zymosan pretreatment; *imm. days*, days between experiment and immunization with Comirnaty; *HVD*, human vaccine dose; *CMT x HVD*, *x*-fold HVD dose of injected Comirnaty; *HSR*, hypersensitivity reaction; *CAS*, cardiopulmonary abnormality score; *described in 2.2.3. 1st and 2nd inj*, CAS after the first and second injection of Comirnaty; *AVA*, anti-vaccine antibody; *anti-PEG*, PEG used as antigen in the ELISA, anti-CMT; Comirnaty was used an antigen in the ELISA; *PAP*, pulmonary arterial pressure; *SAP*, systemic arterial pressure. 11 ANA is the animal undergoing anaphylaxis. The anti-PEG IgM is given as arbitrary units, while the anti-CMT values are relative ODs using pig no. 2 as reference. % PAP, SAP, granulocyte, lymphocyte, and thrombocyte numbers were determined in mmHg, mmHg, 10^9^/mL, 10^9^/mL, 10^6^/mL units, respectively. Pig 5 died during anesthesia due to aspiration. This animal was substituted with pig no. 9, in experiment #11. Hence, there are 14 experiments with pig labels up to 15

#### Summary of experimental variables and physiological changes caused by the vaccine

Beyond the blood pressure and heart rate changes illustrated in Fig. 1, i.v. injection of Comirnaty caused other symptoms of HSRs as well, including skin and blood cell changes. These data are shown in Table 1, which stratifies the 14 pigs used in this study according to treatment variables (zymosan pretreatment, Comirnaty immunization and challenge, and injection order). In addition, the table gives the anti-PEG and anti-vaccine IgM antibody titers before the vaccine challenge.

The data show that 6 pigs (No. 1, 2, 6, 9, 10, and 14) developed more or less HSR to the 1st i.v. bolus of Comirnaty administered in the 1-5x HVD range. The most prominent (and CARPA specific) HSR symptom was pulmonary hypertension with PAP rise to 120–300% of baseline. However, as illustrated in Fig. 1B and C, the second injection of 2x and 5x HVD of Comirnaty failed to cause hemodynamic changes, implying tachyphylaxis. We observed skin flushing in 2 pigs, and 10–60% blood cell changes in most animals, including granulopenia followed by granulocytosis, lymphopenia, and thrombocytopenia. Among these blood cell changes, the most consistent was lymphopenia, in 79% of the animals, followed by granulocytosis, in 64%. The latter was preceded by granulopenia and thrombocytopenia in 36% of pigs. These blood cell changes did not correlate with the hemodynamic or other changes, suggesting independent pathomechanism.

A further preliminary conclusion suggested by the data in Table 1 is that these reactions did not depend on the body weight and sex of animals, immune preconditioning with zymosan, or immunization with the vaccine i.m. or i.v. Among these experiments, one particularly stood out because the pig underwent anaphylaxis, as detailed below.

#### Anaphylaxis caused by Comirnaty

**Fig. 2:**
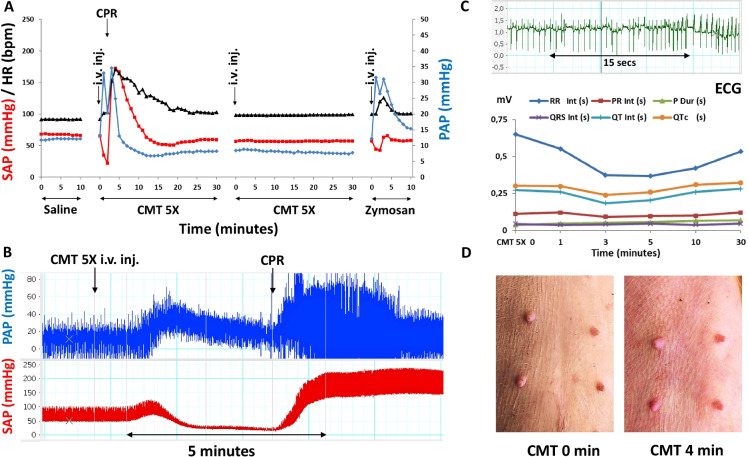
Anaphylaxis with tachyphylaxis in a pig (no. 9) injected with 5x HVD. Similar experiment and abbreviations as used in Fig. 1A–C. **A** Mean PAP (blue), SAP (red), and HR (black) during the whole experiment. **B** Real-time pulse pressure recording of the reaction during the initial 10 min. **C** top: a 25 s ECG recording during the reaction showing arrhythmia, and bottom: changes of ECG parameters after the first injection of 5x Comirnaty up to 30 min, wherein RR int, PR int, QRS int, QT int. mean the RR, PR, QRS, and QT intervals, and P dur and QTc mean duration, in seconds. The latter values were obtained during 15 s analysis of the ECG at the indicated times. **D** Digitalized photographs of baseline (CMT 0) and skin flushing caused by Comirnaty at 4 min (CMT 4 min) after i.v. injection

Figure 2 shows different signs of an anaphylactic reaction that we observed in one of the 6 reacting pigs (no. 9) injected with 5x HVD Comirnaty. For no predicted reason this animal fell into shock within 2 min after injection of Comirnaty. The sudden decline of mean SAP to < 20 mmHg is associated with compensatory tachycardia and extensive, biphasic PAP wherein the wave’s splitting can be explained with superposition of sharply declining SAP on the rise of PAP (Fig. 2A). Resuscitation with epinephrine and cardiac massage led initially to a transient hypertensive overshoot, but after 20–25 min, all hemodynamic parameters returned to near normal. After a few minutes of stabilization, when the animal was injected a second time with the same dose of Comirnaty, there was full tachyphylaxis, just as in Fig. 1B. The final reaction to 0.1 mg/kg zymosan was also smaller than the first reaction, although on a weight basis, zymosan was applied at ~24-fold higher amount than the mRNA content in 5x HVD Comirnaty, and ~ 8-times higher than the DSPC content in 5x HVD (based on Table 2). Figure 2B shows real-time blood pressure recordings in the initial 10 min highlighting that the progress of shock entailed the narrowing of pulse pressure, i.e., a more expressed reduction of the systolic than the diastolic blood pressure relative to baseline. These changes were also associated with the EKG abnormalities (reduction of RR intervals and arrhythmia) (Fig. 2C) and skin flushing (Fig. 2D).

#### The dose dependence of Comirnaty’s pulmonary vasoactivity and its comparison with Doxil

Going back to Fig. 1, the 1x HVD of Comirnaty caused small, but clearly discernible pulmonary vasoactivity, while the 2x and 5x HVD doses led to maximal responses in terms of first-injection PAP peak height (Fig. 1A-C). Based on this reaction parameter and these pilot tests in 3 pigs, the minimal and maximal reactogenic doses, and hence, the dynamic window of PAP response to Comirnaty, are within 1-2x HVD of Comirnaty. However, it should be remembered that the majority of animals (eight) did not show any pulmonary reaction and that the height of the PAP response is only one parameter of HSR, whose different symptoms may have different dose-dependence. Nevertheless, this peak height can be used to compare the pseudoallergic reactogenicity of different liposomal and other drugs, as it is a highly sensitive and relatively reproducible quantitative endpoint of cardiopulmonary distress [28].

#### Comparison of the pulmonary vasoactivities of Comirnaty and Doxil

**Fig. 3:**
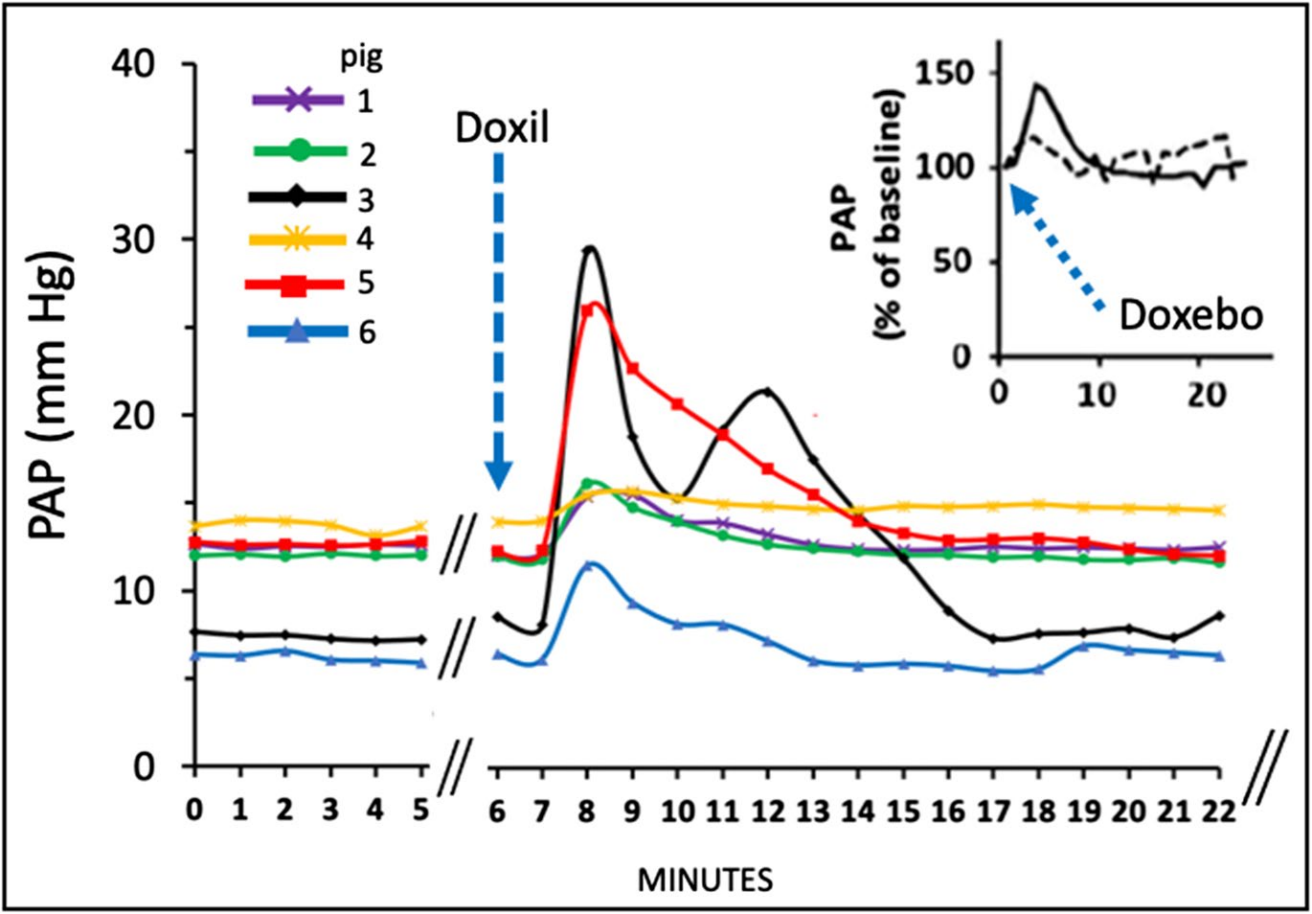
Pulmonary hypertension caused by Doxil in 6 pigs, each curve representing a different animal. The averaged PAP data in Fig. 2A of ref. [21] were modified and replotted to illustrate the individual variation in the progress of pulmonary hypertension after i.v. injection of 0.1 mg phospholipid/kg Doxil. The insert shows essentially similar effect of 0.1 mg phospholipid/kg Doxebo, i.e., doxorubicin-free placebo Doxil, *n* = 2, showing that the effect was due to the liposomes

The above information led us to use data from an earlier pig study [21], where we tested the reactogenicity of PEGylated liposomal doxorubicin (Doxil) [29], the first FDA-approved nano-drug whose HSR-inducing effect has been known since its introduction into cancer chemotherapy in 1995 [30]. The study showed similarly small PAP peaks and runaway maximal reactions after i.v. injection of 0.1 mg/kg of liposomal phospholipid (Fig. 3), suggesting that the pulmonary vasoactivity of the vaccine and Doxil have a common underlying mechanism in pigs and that the above minimally reactive doses can be taken as being roughly equipotent in pulmonary reactivity.

Based on the above findings and considerations, we compared the absolute amounts of common ingredients in these minimally reactogenic doses of the vaccine and the liposome, namely, the amounts of injected phospholipids and PEGylated lipids related to pig body weight. The comparison (Table 2) showed ~38- and ~17-fold less total phospholipid and PEGylated lipid in the vaccine compared to Doxil, respectively. The cargos (mRNA vs. doxorubicin), cholesterol, and total lipid amounts were also much lower in the vaccine (~18-, ~4-, and approximately sixfold less, respectively, Table 2), which implies stronger reactogenicity of the vaccine than Doxil, at least in causing minimal pulmonary hypertension in pigs. It should be emphasized that pulmonary hypertension in pigs is just one symptom of HSRs, thus the above comparison under special experimental conditions has no relevance to other effects or toxicities of Comirnaty and Doxil.

**Table. 2:** Comparison of ingredient amounts in Comirnaty and Doxil, causing minimal pulmonary hypertension in pigs. Values normalized to pig weight

Ingredients	Comirnaty	Doxil	Dox/Comirnaty ratios^1^
	μg/kg*		
Cargo^2^	0.87	15.66	18.00
Phospholipid^3^	2.61	75.0	28.73
Ionizable lipid^3^	12.5		N/A
PEGylated lipid^3^	1.45	25.0	17.24
Cholesterol^3^	5.81	25.0	4.30
Total phospholipid^3^	2.62	100.0	38.17
Total lipid^3^	22.38	125.0	5.58

^*^Entries are absolute amounts of ingredients in the minimal reactogenic i.v. bolus doses of Comirnaty (CMT, 1x HVD) and Doxil (0.1 mg phospholipid/kg) in pigs, divided by the average weight of animals (34.4 kg, Table 1). The indexed entries are: ^1^Doxil/Comirnaty absolute weight ratios in their minimal reactogenic dose calculated for the specified ingredients; ^2^the absolute amounts of ingredients in 1x HVD (0.3 mL) Comirnaty are as follows: Cargo: 30 μg mRNA; phospholipid: 90 μg 1,2-Distearoyl-sn-glycero-3-phosphocholine (DSPC); ionizable lipid (ALC-0315): 430 μg (4-hydroxybutyl-azanediyl) bis (hexane-6,1-diyl)bis(2-hexyldecanoate); PEGylated lipid (ALC-0159): 50 µg polyethylene glycol)-2000]-N,N-ditetradecylacetamide; cholesterol: 200 μg. The corresponding ingredients in Doxil containing 0.1 mg/kg phospholipid are: 16 μg doxorubicin; 75 μg fully hydrogenated soy phosphatidylcholine (HSPC); 25 μg N-(carbonyl-methoxypolyethylene glycol 2000)-1,2-distearoyl-sn-glycero-3-phosphoethanolamine sodium salt (MPEG-DSPE); 25 μg cholesterol. These ingredient data were obtained from the prescribing information for both agents [19, 27]^1^

### The effects of i.v. Comirnaty on plasma TXB2 in pigs

The vascular effects of thromboxane A2 (TXA2) explain the pulmonary hypertensive effect of liposome-induced C activation, and TXB2 is a stable byproduct of TXA2 metabolism whose plasma level was shown previously to closely parallel the hemodynamic changes caused by liposomes in pigs [31]. Thus, we measured this analyte in the plasma of pigs treated with Comirnaty.

**Fig. 4:**
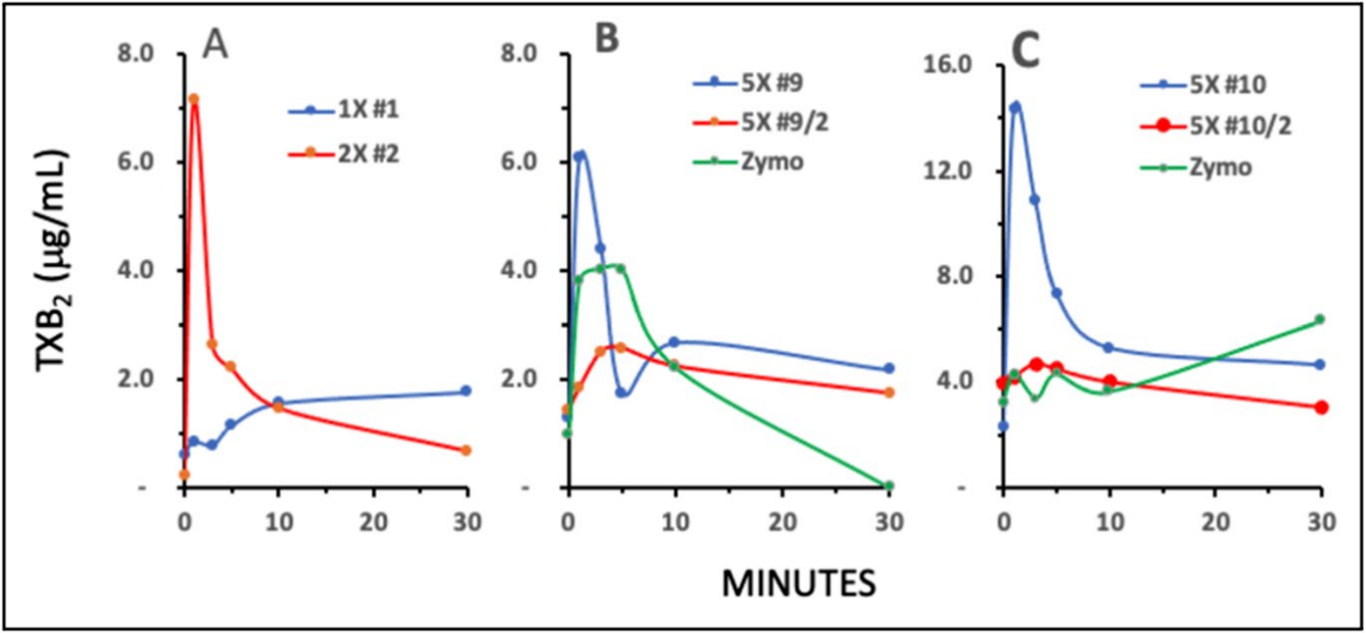
Plasma TXB_2_ levels in pigs #1 and #2 following i.v. injection of different doses of Comirnaty, whose hemodynamic changes are shown in Fig. 1A and B. In B and C, 9/2 and 10/2 mean second injection of the same dose

Figure 4A shows the time course of TXB2 rises in the plasma of pigs in Fig. 1A and B. In remarkable parallelism with the PAP changes, the animal injected with 1 × HVD (pigs #1, blue) displayed minimal, while the one injected with 2 × HVD (pigs #2, red) displayed immediate, maximal rise of TXB2 on the same time course as the hemodynamic changes occurred. Figure 4B shows another parallelism with the hemodynamic changes: animal no. 9, which underwent anaphylaxis, displayed a sudden, major rise of TXB2 (5 × #9, blue), while the second dose which caused no rise of PAP caused only minimal rise of TXB2 (5 × N#9/2, red). This panel also shows the effect of zymosan (green), also rising in parallel with pulmonary hypertension, but in a more extended fashion. Yet a 3rd pig in this series, which also developed maximal pulmonary reaction with tachyphylaxis after the first but not the second injection, reproduced the same TXB2 profile as pig #9. These data provide further evidence for the similar, thromboxane A2-dependent mechanism of Comirnaty and PEGylated liposome-induced pulmonary hypertension in pigs.

### Other physiological parameters measured

Among the other physiological parameters measured (RBC, Hgb, ECG wave intervals and amplitudes, SPO2, respiration rate, Et.CO_2_, core temperature, none showed consistent, biologically relevant changes, except in Exp # 11 (pig no. 9), the animal undergoing anaphylaxis, displayed decreased respiratory rate and etCO_2_, along with ECG signs of hypoxia and arrhythmia (Fig. 2).

### The complement activating effect of Comirnaty in pig serum

**Fig. 5:**
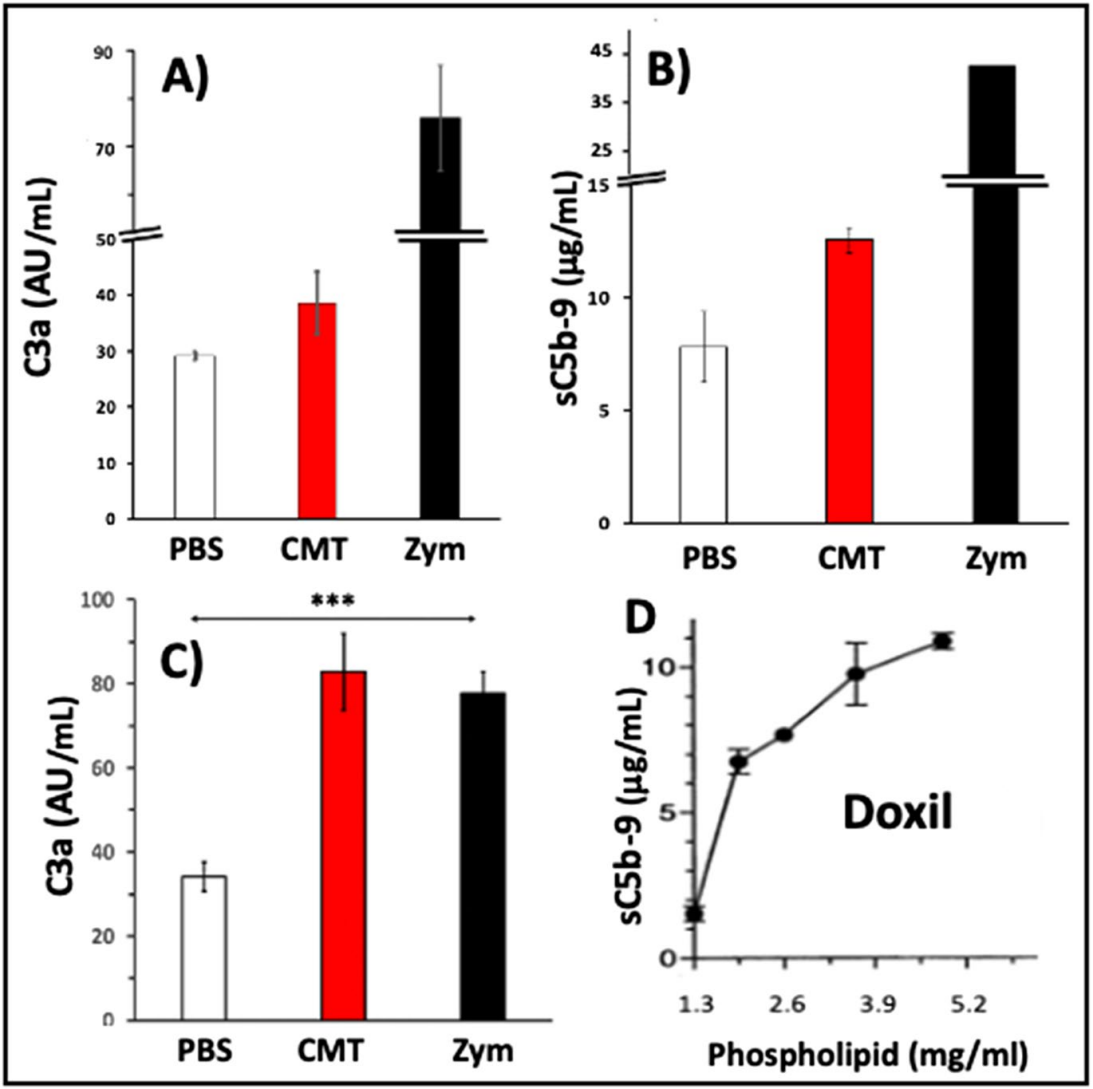
Complement activation by Comirnaty (CMT) in pig serum in vitro*.* **A, B** Healthy pigs’ sera (*n* = 3) were incubated with Comirnaty at 37 °C for 60 min at a final vaccine mRNA and phospholipid (DSPC) concentrations of 20 and 60 μg/mL, respectively. Panel **A** shows the C3a, and **B** the sC5b-9 levels in the same samples. Zymosan was applied at 0.1 mg/mL. **C**, similar measurement as in **A** and **B**, except that the vaccine concentration was doubled (mRNA and total phospholipid were 40 and 120 μg/mL, respectively) and we measured only C3a as endpoint. Bars are mean + / − SE, *n* = 5. ***, *P* < 0.005 by ANOVA. **D** is a reproduction of Fig. 2B in ref. [32] showing the dose dependence of C activation by Doxil in human serum in vitro

Figure 5 shows two experiments wherein we measured C activation by Comirnaty in pig serum at two vaccine concentrations, using porcine C3a (Fig. 5A, C) and sC5b-9 (Fig. 5B) as endpoints of C activation. These data show significant dose-dependent C activation, the effect of the higher dose being comparable to that of zymosan (Fig. 5C). Thus, the observation on similar or greater hemodynamic abnormalities caused by 5x HVD Comirnaty (4.4 µg/kg mRNA and 13.2 µg/kg DSPC), compared to zymosan (100 µg/kg) in pigs (Figs. 1C and Fig. 2) has been reproduced in a serum C assay in vitro under entirely different conditions. This represents an indirect support for the causal role of C activation in HSRs, i.e., CARPA.

It is also important to note that the stronger (than zymosan) C activating effect of Comirnaty (Fig. 5C) was observed at 120 μg/mL DSPC, which is about 17 × lower than the minimal C activating effect of Doxil phospholipids (HSPC + MPEG-DSPE) in human serum (~ 2 mg/mL, Fig. 5D). These proportions are also comparable to the phospholipid and PEGylated lipid ratios in the minimal pulmonary reactogenic doses of Doxil and Comirnaty in pigs (~ 29 and 18, Table 2).

### Non-mRNA-coded immunogenicity of Comirnaty and its impact on HSRs

#### The immunogenicity of polyethylene glycol (PEG) in Comirnaty

The last 6 animals in this study were immunized with 1x HVD Comirnaty either i.m. or i.v., and the blood anti-PEG IgM levels were periodically determined until the 5x HVD Comirnaty challenge 14–20 days after immunization (Table 1). Five of these animals showed no or minimal HSR, while 1 animal (no. 9), immunized i.v. 14 days earlier, fell into anaphylactic shock (Fig. 2).

**Fig. 6:**
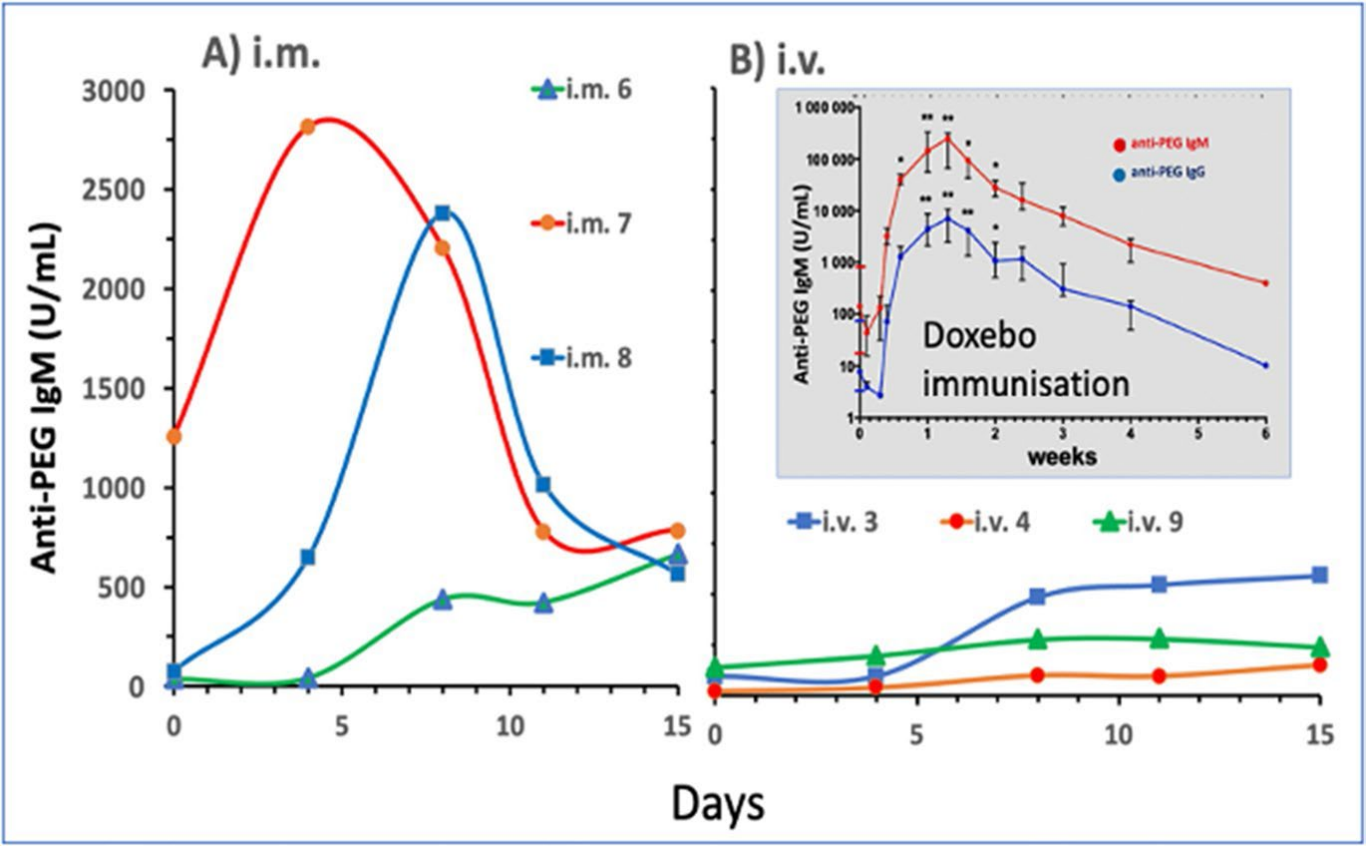
Time course of the rise of blood anti-PEG IgM in pigs immunized with the HVD of Comirnaty either i.m (A) or i.v. (B) 2 weeks before i.v. administration of 5x Comirnaty. The insert in Panel B, showing the anti-PEG IgM and IgG titers in pigs immunized with Doxebo, is reproduced from ref. [21]. It shows hugely more effective and long-lasting immunogenicity of i.v. Doxebo than the vaccine

Figure 6A and B show the rises of anti-PEG IgM in these 2 groups. I.m. immunization (Fig. 6A) was more effective than the i.v. procedure, resulting in 5-6-fold higher peaks in 2 of 3 pigs than the peak heights in all 3 i.v.-immunized animals (Fig. 6B). Antibody formation reached the maximum in the 3–8 days window and then declined after 10–12 days. These findings suggest that Comirnaty can induce anti-PEG IgM, but this effect is relatively weak and short, it cannot be involved in Comirnaty-induced HSRs >2 weeks after immunization.

The insert in Fig. 6B shows the immunogenicity of 0.1 mg/kg Doxebo in pigs, yielding orders of magnitude higher anti-PEG IgM titers than we got from Comirnaty, which also lasted for several weeks [21]. All these Doxebo-immunized pigs underwent anaphylactic shock identical to the one seen in the present study [21], so a causal role of anti-PEG IgM in Comirnaty-induced HSRs cannot be a priori excluded. It is possible that the 17-fold lower amount of PEG in the vaccine relative to that in Doxebo (Table 2) and missing the antibody peaks excluded seeing the anaphylactogenicity of anti-PEG IgM in the present study. This also means that the anaphylaxis of pig no. 9, which was i.v. immunized with Comirnaty, could not be explained with anti-PEG IgM, highlighting the likely involvement of other antibodies, and/or a C-independent mechanisms of anaphylaxis, as suggested by the double-hit theory (see Discussion).

#### The non-mRNA-coded immunogenicity of the whole Comirnaty LNP

**Fig. 7:**
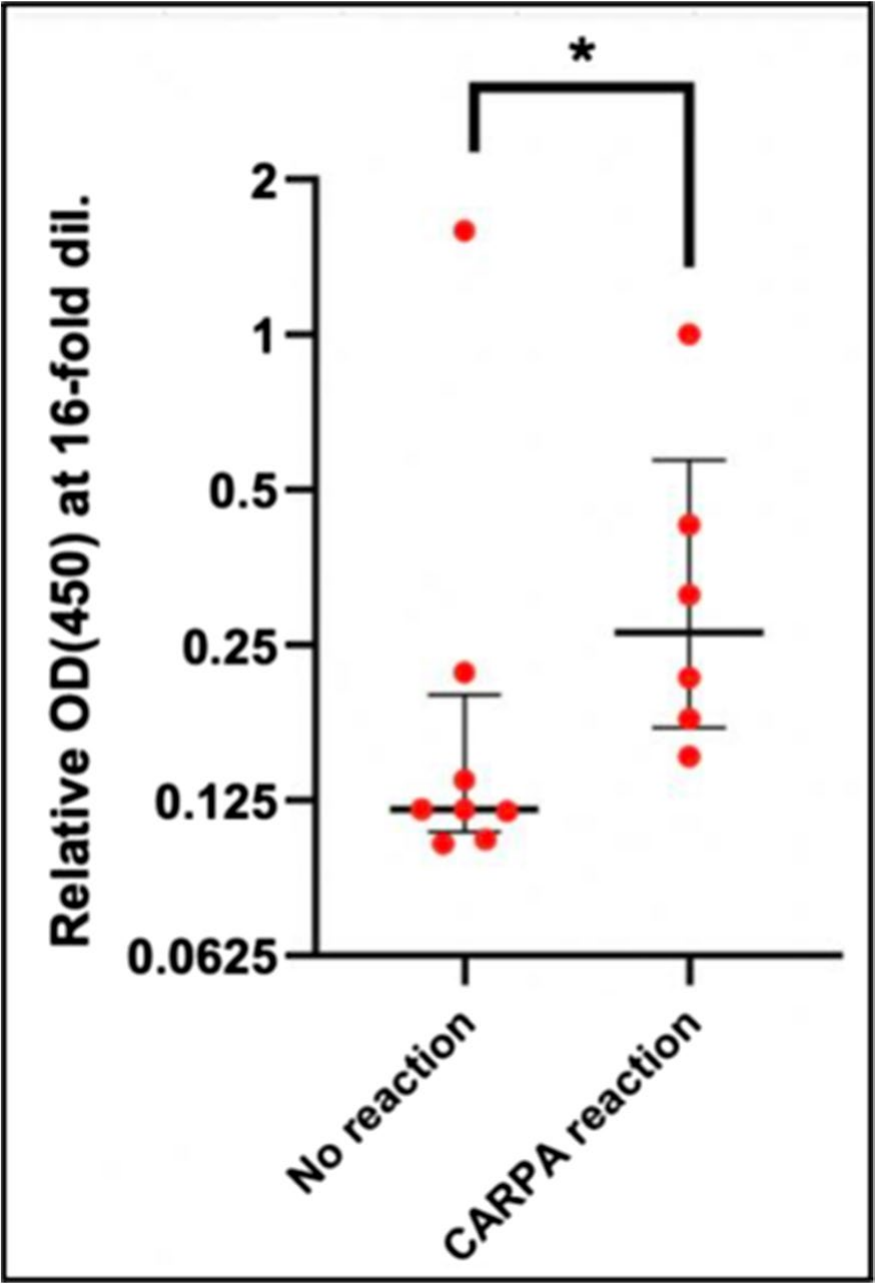
Anti-Comirnaty LNP IgM levels (median ± IQR) in pigs before i.v. injection of 1-5 HVD of Comirnaty. The *Y* values are relative to the ELISA OD values obtained in pig no. 2, wherein 2x HVD caused maximal pulmonary response. The group “CARPA reaction” on the x-axis includes all reactive animals regardless of trigger vaccine dose. *, *P* < 0.02

In addition to the rise of anti-PEG IgM after immunization of the animals with Comirnaty, we also measured the pre-challenge blood levels of anti-Comirnaty LNP IgM by a modified ELISA, wherein the antigen used to coat the ELISA plates was the whole vaccine LNP rather than PEG. As shown in Fig. 7, the average anti-CMT IgM level was significantly higher in the animals displaying HSR compared to nonreactors, which suggests a contribution to the HSRs of natural or induced IgM reacting with one or more vaccine components. Here again, the lack of outstanding anti-LNP IgM response in pig no. 9 argued against the sole role of these (relatively low-level) IgMs in anaphylaxis induction.

## Discussion

### Allergic reactions and anaphylaxis to LNP-mRNA vaccines: an unsolved hurdle of COVID-19 vaccination for a few

Anaphylaxis is a very rare complication of vaccinations, occurring in about 1.4 cases out of a million immunizations worldwide (14). Focusing only on the LNP-mRNA based COVID-19 vaccines, this rate seems to be surpassed by a factor that varies in different estimations (see below), but considering the effective prevention and treatment measures of anaphylaxis today (1% mortality rate [33] or less), the clinical significance of the phenomenon is very small. Yet, due to the crucial role of vaccinations in halting the COVID-19 pandemic, the rare HSRs and anaphylaxis cases obtain substantial regulatory, scientific and public attention [2, 3, 5–9, 14, 34–36]. Signs of unceasing concern about allergic reactions to the vaccine include multiple questions on allergies on consent forms with the exclusion of people with severe allergies to any of the vaccine components, history of anaphylaxis, autoimmune diseases, Guillain-Barré syndrome, Bell’s palsy, and cosmetic dermal filler recipients [14, 37]; the mandated 30-min post-vaccination monitoring for everyone; the directives that vaccination centers must be prepared for emergency treatment of anaphylaxis or other forms of allergic reactions; guidance on how to prevent and treat vaccine-induced anaphylaxis [2, 9]. Most recently, a multicenter clinical trial started to establish the incidence of HSRs to LNP-mRNA-based vaccines in a high-allergy/mast cell disorder population in comparison to a representative population without severe allergies or mast cell disorders [38].

### The relative risk of anaphylaxis and severe allergic reactions to LNP-mRNA vaccines: variations of prevalence data

Due to the heightened alert and preventive measures, the rate of allergic reactions to LNP-mRNA vaccines tends to decrease, but case reports continue to emerge in the VAERS (see statistical methods). The most recent official (Center of Disease Control, CDC) estimate is 2.5–4.7 anaphylaxis per million vaccinations with Comirnaty [8, 39], while our analysis of the latest (Oct, 2021) data in VAERS, performed with attention not to overestimate the rate (see Methods) gave 15.7 and 16.3 anaphylaxis per million vaccinations with the Pfizer/BioNTech and Moderna vaccines after roughly 242 and 154 million US recipients of these vaccines, respectively. Other recent studies found 1 anaphylaxis in 4,000 vaccinations (250/million) [4] and 22 in 38,895 (566/million, ~ 1 in 1,800 vaccinations) [10]. Counting not only anaphylaxis but all more or less severe HSRs to Comirnaty gave 10 HSRs in 2000 vaccinations (~1 in 200, 0.5%) [11], which rate actually represents a typical prevalence of infusion reactions to PEGylated nano drugs [40]. The most frequent adverse symptoms of vaccinations in the study of Gringeri et al. [11] were airway obstruction, laryngeal edema, hypotension, tachy- or bradycardia, urticaria, and asthma, which are also prevalent symptoms of CARPA caused by i.v. nano drugs [13].

### Concepts on the mechanism of anaphylaxis to LNP-mRNA vaccines: CARPA and others

In absence of dedicated human or animal studies, explanations for the mechanism of LNP-mRNA-induced HSRs are hypothetical to date. Although classical, IgE-mediated type I hypersensitivity to a vaccine component can occur, it cannot account for all HSRs since most people inflicted with HSR show no positive skin test for the vaccine or its components. This also applies to PEG, the only component of LNP-mRNA vaccines whose widespread presence in foods, drinks, medicines, cosmetics, toothpaste, shampoos, sunscreens, and many other commodities of routine life [40] could easily explain sensitization. Skin test positive allergy to PEG does exist, but it is a rare, severe condition of which most patients are well aware [3, 41–43]. They are excluded from getting PEG-containing vaccines.

Accordingly, the large majority of HSRs to PEG-containing vaccines are among people who do not respond to PEG via IgE-mediated allergy but via an alternative mechanism called pseudoallergy, of which most people are not aware until they develop symptoms. Also consistent with pseudoallergy, Kranz et al. reported no or only mild reactions after the second Comirnaty boost [4, 44], which implies tachyphylaxis, a characteristic feature of C-mediated pseudoallergy, i.e., CARPA [45].

The likely involvement of CARPA in LNP-mRNA vaccines was proposed at an expert meeting convened by the National Institute of Allergy and Infection Diseases in December 2020 in response to the anaphylaxis cases in the UK after the first immunizations with Comirnaty [46]. The idea stemmed from the similarity of the time course and symptoms of vaccine-induced HSRs to those seen during infusion reactions to PEGylated liposomes (Doxil) [13, 47, 48], to which Comirnaty resembles. In fact, a study published shortly before the pandemic showed anaphylactic shock to a small i.v. dose of Doxil in pigs sensitized to anti-PEG antibody-induced HSRs by i.v. immunization with PEGylated liposomes (Doxebo) [21]. This reaction was shown to be C-mediated [21], providing experimentally derived evidence for CARPA underlying such “pseudo-anaphylaxis.”

Beyond CARPA, there are other explanations for LNP-induced anaphylactic reactions. One theory attributed the phenomenon to “the relatively high number of local immune cells at the site of injection “getting too much excitement” due to “technically delivering of an adjuvant” [46]. The problem with this explanation is that the LNP-mRNA vaccines do not contain adjuvants technically, and that it fails to explain how a local immune stimulus can trigger systemic symptoms, including anaphylaxis. C activation was ruled out on basis that it is expected to “proceed in almost all vaccine recipients, but anaphylaxis is very rare” [49]. However, this simplistic connecting of C activation to anaphylaxis can also be questioned as it neglects that C activation feeds into HSRs and anaphylaxis via several signaling channels, each having multiple relay steps and controlling factors [21]. This complexity of molecular and cellular interactions renders the causality between these phenomena non-linear, individual threshold-dependent [50], highly variable. As discussed later for the “double hit” hypothesis, only overwhelming C activation may be rate-limiting, the primary cause of anaphylaxis. In most cases, the role of C activation may be to provide essential co-stimulation for vasoactive mediator release [51, 52] by allergy mediating immune cells [21].

Yet, a further mechanism considered for vaccine-induced anaphylaxis is “immunization stress-related response,” a newly defined adverse effect following immunizations [53] with which anaphylaxis can be misdiagnosed and vice versa [53–57]. Among other symptoms, sudden hypertension may raise uncertainties, which is typical of both pseudoallergy and anxiety, but not of anaphylaxis.

### Historic support for the involvement of C activation in the HSRs to LNP-mRNA vaccines

Complement activation entailing anaphylatoxin release, as a potential contributing cause of HSRs and anaphylaxis was proposed by Hugli et al. exactly 40 years ago [58]. Since then, several lines of evidence attested to this mechanism of anaphylaxis [59], including the finding that C depletion and production of C3a and C5a in human anaphylaxis correlated with the severity of the reactions, and the induction of immediate wheal-and-flare reactions after intradermal injection of anaphylatoxins in healthy volunteers [52, 60–62]. The minimal amount of C3a, C4a, and C5a that induced the latter changes were 10 pmol, 1 nmol, and 40 fmol, respectively [52], implying stronger reactogenicity than that of histamine. These reactions were dose-dependent and reached maximum at 5-10 min after injection, which is a typical time course of anaphylaxis, too. Using the pig model described here, the causal role of anaphylatoxins in NP-induced HSRs was described in 1998-99 [15, 63], and since then, several lines of experimental evidence have proven the concept [18, 64, 65].

### Experimental support for the involvement of C activation in HSRs to LNP-mRNA vaccines in the present study

This study provided two convergent lines of evidence for CARPA underlying the HSRs to Comirnaty. One is the rise of typical CARPA symptoms in pigs after i.v. administration of the vaccine, and the second is that Comirnaty can activate C in porcine serum in vitro.

#### Induction of CARPA symptoms by Comirnaty *in vivo*

It has been described in numerous previous publications that intravenous injection of liposomes and other NPs can trigger a unique tetrad of acute hemodynamic, hematological, skin, and biochemical changes including pulmonary hypertension, systemic hyper- or hypotension, tachy- or bradycardia with arrhythmia, granulopenia followed by granulocytosis, lymphopenia, thrombocytopenia, flushing, rash, and rises of plasma thromboxane B2, PAF, sC5b-9, leukotriene B2, and some others [15–18]. These physiological changes were reproduced by i.v. injection of Comirnaty in 6 pigs out of 14 with one anaphylaxis. Although the expressions of most symptoms were minor and transient, clinically inconsequential (except for anaphylaxis), their synchroneity, consistent direction, comparable duration, and association with rises of plasma thromboxane and anti-Comirnaty IgM provide a clear fingerprint of the CARPA tetrad, leaving no doubt about the model’s utility to study CARPA as a possible mechanism of HSRs to Comirnaty. However, we do not know why some pigs did and others did not show any physiological change in response to Comirnaty injections, so the factors enabling or disabling the reactions remain to be established.

#### Complement activation by Comirnaty

We found that Comirnaty is a strong C activator in pig serum at a concentration that may be relevant in vivo, as delineated in the next section. Here we address the mechanism of activation, which, in theory, can proceed via all three activation pathways. Namely, classical pathway activation may occur as a consequence of the binding of natural (or induced) antibodies to the phospholipid, cholesterol, and PEG molecules on the LNPs [21, 40, 66, 67], as well as the binding of C1q to the mRNA [68]. The positively charged (ionizable) lipids can activate C via the alternative pathway [69, 70], while the transcribed spike (S) protein can activate the lectin pathway [71]. Because the LNP-mRNA in Comirnaty is unstable at body temperature, and because the sheer stress upon injection may also cause the breakdown of some LNP-mRNAs [72], it is possible that disintegration of the LNPs could enhance C activation via increased exposure of the mRNA and/or other activating surfaces to C proteins.

The fact that PEGylated, mRNA containing LNPs can activate C is not new; it was described, among others, in human serum in vitro [73] and in rats and monkeys in vivo [74].

Despite this prior knowledge on C activation by nucleic acid-containing liposomes, C activation by Comirnaty, or any other COVID-19 vaccine, has to our best knowledge, not been described in the literature, although it may be vital for the vaccine’s immune-stimulatory action, and, hence, efficacy.

### The relevance of Comirnaty reactions in pigs to the human vaccine reactions

Apart from the shock symptoms that represent a clear indication of the utility of the pig model to study the human anaphylaxis to Comirnaty, the fact that we injected multiple HVDs of Comirnaty i.v. instead of 1x HVD i.m. rightfully questions the human relevance of our data. The hypotheses below represent an attempt to reconcile the above dissimilarities and highlight how our observation could explain the human HSRs to the vaccine.

#### Opportunities for C activation by Comirnaty in vaccinated humans

In humans undergoing vaccinations with Comirnaty, C activation by the vaccine, if it occurs, could take place either locally, at the site of injection, and/or systematically, in plasma, after the vaccine or its disintegrated components get into the blood.

As for local activation, this possibility might appear counter-intuitive, since C activation is generally perceived as a chain reaction in plasma with all C proteins being present. However, as an inflammatory phenomenon, C activation might also occur in inflamed tissues, and the erythema, warmth, swelling, pain, induration, and/or tenderness at the injection site and its vicinity, which are signs of an inflammatory response, are frequent side effects of vaccinations [75, 76]. The process involves the accumulation of C proteins in the swollen tissue, partly from the inflammatory exudate which is rich in C proteins, and partly from the activated mast- and dendritic cells and infiltrating immune cells (lymphocytes, monocytes, and macrophages), which release C3, C5, factors B and D beside many other proinflammatory molecules [77]. Thus, the vaccine’s intrinsic capability for immune stimulation may entail the accumulation of C proteins at and around the site of vaccinations.

Whether or not they could be activated by the vaccine NPs, the fundamental finding in the present study that Comirnaty is a strong C activator in pig serum gives a “most likely” answer. These in vitro C activation experiments showed that the vaccine activates C at a phospholipid (DSPC) concentration of already 60 µg/mL (Fig. 5A). The phospholipid concentration in the injected 0.3 mL Comirnaty dose is 0.3 mg/mL, 5-fold above the above threshold of C activation in serum, and, as discussed above-, DSPC is not the only component in the vaccine that can activate C. Obviously, the initial vaccine NP concentration declines as the vaccine bolus gradually mix with the inflammatory exudate and the NPs bind to immune cells or exit via the lymph or blood vessels, but C activation could already start and reach a sufficient extent to produce reactogenic amounts of anaphylatoxins.

Beyond the local activation, the finding that the vaccine is a strong C activator raises the possibility that it also activates C in the blood, once the NPs exit the site of injection as delineated below.

#### Possible exit routes of LNPs and anaphylatoxins from the injection site into the circulation

As discussed above, the vaccine can induce an inflammatory reaction at the site of its injection. The associated increase in capillary permeability allows the transcapillary passage of ~ 100 nm NPs, a known precondition of the enhanced permeability and retention (EPR) phenomenon utilized in the therapy of cancer and inflammatory diseases with liposomal drugs [30, 78–80]. Considering the size of Comirnaty NPs (80–100 nm) [72], the reverse of EPR may occur allowing the vaccine NPs to get out from the tissue into the capillary bed. Then, the blood’s natural flow leads the way via the muscle venules into the axillary vein. Another exit route is the lymph, percolating the axillary lymph nodes to reach the vena cava via the thoracic duct. The same exit opportunities exist for locally formed anaphylatoxins with the benefit that anaphylatoxins are small glycopolypetides (C5a MW: 11.0–11.5 kDa) whose passage may be much less limited.

#### C activation-independent release of anaphylatoxins

The current paradigm in immunology is that anaphylatoxins are end products of C activation in plasma. However, this perception needs to be updated by the information that anaphylatoxins can get into the blood or intercellular spaces via direct cellular secretion following intracellular proteolysis of parent C proteins by cathepsin L and other proteases [77]. This effect was shown in activated T and other activated immune cells as a mechanism of autocrine and/or paracrine positive feedback amplification of inflammatory response. Taken together, there are numerous redundant, possibly additive mechanisms by which Comirnaty or other similar NPs could lead to anaphylatoxin release into the blood.

#### Can anaphylatoxin activity explain HSRs?

Once in blood, the next question is whether the amount of circulating anaphylatoxins is sufficient for triggering a systemic reaction? It should be remembered in this regard that the anaphylatoxins C3a and C5a are the most potent permeability factors and mast cell degranulation inducers yet described [60], exerting substantial hemodynamic changes in the nM (> 1 ng C5a/mL blood) concentration range [81, 82]. Consistent with this fact, earlier pig studies showed that iv. injection of 10 ng/kg rhuC5a (~0.2 ng/mL plasma) in pigs, despite of species difference, triggered a visible drop in SAP, while a thousand-fold larger dose induced massive pulmonary hypertension with bradyarrhythmia, apnea, and cardiac arrest, requiring resuscitation [20]. The human data on the dose dependence of the skin effects of anaphylatoxins were discussed above, 40 fmole (0.44 ng) C5a being the lowest dose that caused wheel and flare reaction in the skin. Taken together, these data suggest that nano-to-microgram amounts of anaphylatoxins released in blood might contribute to the HSRs and anaphylaxis.

In addition to local activation of C, the finding that the vaccine is a strong C activator raises the possibility that despite substantial dilution in plasma, C activation could also occur after the vaccine NPs reach the blood because of reverse EPR, as described above or accidental injection into a small blood vessel. In the latter case, if the whole amount of the vaccine gets rapidly in blood, the situation corresponds to the response of pig no. 1, which displayed a minor, but a clear reaction to the HVD of Comirnaty.

### Possible explanation of the rarity of vaccine-induced anaphylaxis

We have pointed out previously that C activation may be a co-trigger in certain (but not all) pseudoallergies, it may be the sole cause only in case of overwhelming anaphylatoxin production or extreme sensitivity for anaphylatoxin effects [18, 21, 64, 65, 83–85]. This concept is articulated by the “double hit” hypothesis [13, 16], postulating that pseudo-allergic reactions arise when two or more reaction triggers simultaneously “hit” on immune cells, one being the binding of anaphylatoxins to their receptors and another is the direct binding of another trigger molecule to toll-like and/or other inflammatory surface or intracellular receptors.

Considering the differential secretion of cytokines and other signaling molecules by immune cells in response to different immune stimulants in different people [86, 87], there is already individual variation in the response of blood immune cells to direct activation by PEGylated nano drugs. Likewise, C activation and anaphylatoxin clearance show substantial individual variation. If the intense release of vasoactive allergy mediators (allergomedins) that cause anaphylaxis requires synergistic activation of two or more signal transduction pathways in allergy mediating cells (mast cells, basophils, and macrophages), the rarity of CARPA and occasionality of pseudo-anaphylaxis could be explained with the low chance of coincidental stimulation of synergizing activation pathways. With a stretch of imagination, the C system lives up to its name in the double-hit hypothesis in as much as it “complements” the direct allergen hit on allergy mediating cells, just as it complements the antibodies in their cytotoxic function, motivating Paul Ehrlich in 1899 to call the system “complement” [88].

### The roles of anti-PEG and anti-Comirnaty IgM

We expected that immunization with Comirnaty would accelerate HSRs, just as immunization of pigs with Doxebo accelerated a minor reaction to Doxil into lethal anaphylactic shock due to anti-PEG IgM induction [21]. In fact, the pig that underwent anaphylaxis was immunized with Comirnaty i.v. 2 weeks before, but the absence of similar reaction in the other 2 animals in the same group, and the relatively low anti-PEG IgM (Table 1) in that animal argued against a causal role of anti-PEG IgM in the HSRs under the conditions of the present study. Nevertheless, these data do not exclude a causal or contributing role of anti-PEG antibodies under other conditions, or more effective immunization. Here we used 30-times less PEG than applied in the referred study [21], furthermore, the antigen that we used in the ELISA was not the same PEG conjugate that is present in the vaccine, which may reduce the specificity and sensitivity of the test. Indeed, the ELISA that used the whole vaccine as antigen did show a contributing role of reactive IgM to HSRs, but the titers were still far below the anaphylaxis-inducing anti-PEG IgM levels in our PEGylated liposome study [21]. Based on these preliminary data, low-level antibody-mediated C activation may be a contributing, but not rate-limiting factor in the HSRs to Comirnaty.

### Porcine CARPA as a disease model

The information that Comirnaty, safely administered to hundreds of millions can cause in pigs immune-mediated circulatory and hematological abnormalities, including anaphylaxis, may lead to questioning the suitability of pigs for such studies. However, as stressed previously [18, 65], the porcine “CARPA model” is not a toxicology model; it represents a disease model, that of allergy to i.v administered nanoparticulate drugs and diagnostics, most frequently liposomes. It was used to show the capability of Doxebo [45, 89], an anti-C5a antibody and indomethacin to inhibit these reactions [15] and for developing safe administration protocols for Onpattro [90] and a prednisolone-containing PEGylated liposome [91]. If the porcine CARPA model proves to be valid for studying the HSRs to Comirnaty, it can provide solutions how to prevent this problem hopefully not only for Comirnaty but all other reactogenic drugs and vaccines. Importantly, the recognition that the rare HSRs to mRNA vaccines represent pseudoallergy is on the rise [10, 55, 92, 93].

## Outlook

Because of their rare occurrence, HSRs and anaphylaxis to Comirnaty and other COVID-19 vaccines present a small risk that gives no reason for questioning the overall safety of these vaccines. The worry lies in the individual health and other consequences for those inflicted, potentially in the order of thousands if not tens of thousands worldwide, in light of the large number of immunizations (over a billion) worldwide and high prevalence of allergic (atopic) constitution. These reactions also raise concern on the safety of repeated vaccinations in the face of new variants, due to the potential immunogenicity of the LNP-mRNA construct inducing anti-PEG and perhaps other reactogenic, self-neutralizing antibodies. These considerations call for effective resolution of the problem before it gets more public attention feeding into vaccine hesitancy [94]. Finding a solution in the pig model described here may help to prevent this problem.

The strong C activation by Comirnaty was an unexpected finding in this study in the face of immense attention to the various immune effects of this vaccine. However, it should not be viewed only as undesirable, as C activation may be an essential contributor to the antiviral immunogenicity of Comirnaty, just as it is for other vaccines [95–98]. Complement is known to bridge innate and specific immunity [99, 100], thus, beyond providing first-line defense against the virus and causing harmful immune reactions, it can also be indispensable for efficient long-term immune protection. It will be intriguing to learn about all these different facets of the C system in the context of SARS-COV-2, Comirnaty, and other vaccines.

## References

[1] Polack FP, Thomas SJ, Kitchin N, Absalon J, Gurtman A, Lockhart S, Perez JL, Perez Marc G, Moreira ED, Zerbini C, Bailey R, Swanson KA, Roychoudhury S, Koury K, Li P, Kalina WV, Cooper D, Frenck RW, Hammitt LL, Tureci O, Nell H, Schaefer A, Unal S, Tresnan DB, Mather S, Dormitzer PR, Sahin U, Jansen KU, Gruber WC, Group CCT (2020). Safety and efficacy of the BNT162b2 mRNA Covid-19 vaccine. N Engl J Med.

[2] CDC. Interim considerations: preparing for the potential management of anaphylaxis at COVID-19 vaccination sites summary. https://www.cdc.gov/vaccines/covid-19/clinical-considerations/managing-anaphylaxis.html. Accessed 19 Dec 2021

[3] Garvey LH, Nasser S. Anaphylaxis to the first COVID-19 vaccine: is polyethylene glycol (PEG) the culprit? Br J Anaesth. 2021;126(3):e106ee132 10.1016/j.bja.2020.12.020 PMC7834677 33386124

[4] Krantz MS, Bruusgaard-Mouritsen MA, Koo G, Phillips EJ, Stone CA, Jr., Garvey LH. Anaphylaxis to the first dose of mRNA SARS-CoV-2 vaccines: don't give up on the second dose! Allergy. 2021;76(9):2916–2920. 10.1111/all.14958 PMC8222898 34028041

[5] Hashimoto T, Ozaki A, Bhandari D, Sawano T, Sah R, Tanimoto T. High anaphylaxis rates following vaccination with the Pfizer BNT162b2 mRNA vaccine against COVID-19 in Japanese health care workers; a secondary analysis of initial post-approval safety data. J Travel Med. 2021. 10.1093/jtm/taab090 PMC8344519 34128049

[6] Sellaturay P, Nasser S, Islam S, Gurugama P, Ewan PW (2021). Polyethylene glycol (PEG) is a cause of anaphylaxis to the Pfizer/BioNTech mRNA COVID-19 vaccine. Clin Exp Allergy.

[7] Risma KA, Edwards KM, Hummell DS, Little FF, Norton AE, Stallings A, Wood RA, Milner JD. Potential mechanisms of anaphylaxis to COVID-19 mRNA vaccines. J Allergy Clin Immunol. 2021. 10.1016/j.jaci.2021.04.002 PMC8056854 33857566

[8] Shimabukuro T, Nair N. Allergic reactions including anaphylaxis after receipt of the first dose of Pfizer-BioNTech COVID-19 vaccine. JAMA. 2021. 10.1001/jama.2021.0600 PMC8892260 33475702

[9] Team CC-R, Food, Drug A (2021). Allergic reactions including anaphylaxis after receipt of the first dose of Moderna COVID-19 vaccine - United States, December 21, 2020-January 10, 2021. MMWR Morb Mortal Wkly Rep.

[10] Warren CM, Snow TT, Lee AS, Shah MM, Heider A, Blomkalns A, Betts B, Buzzanco AS, Gonzalez J, Chinthrajah RS, Do E, Chang I, Dunham D, Lee G, O'Hara R, Park H, Shamji MH, Schilling L, Sindher SB, Sisodiya D, Smith E, Tsai M, Galli SJ, Akdis C, Nadeau KC (2021). Assessment of allergic and anaphylactic reactions to mRNA COVID-19 vaccines with confirmatory testing in a US regional health system. JAMA Netw Open.

[11] Gringeri M, Mosini G, Battini V, Cammarata G, Guarnieri G, Carnovale C, Clementi E, Radice S (2021). Preliminary evidence on the safety profile of BNT162b2 (Comirnaty): new insights from data analysis in EudraVigilance and adverse reaction reports from an Italian health facility. Hum Vaccin Immunother.

[12] Mathioudakis AG, Ghrew M, Ustianowski A, Ahmad S, Borrow R, Papavasileiou LP, Petrakis D, Bakerly ND. Self-reported real-world safety and reactogenicity of COVID-19 vaccines: a vaccine recipient survey. Life. 2021;11(3):249. 10.3390/life11030249 10.3390/life11030249 PMC8002738 33803014

[13] Szebeni J (2014). Complement activation-related pseudoallergy: a stress reaction in blood triggered by nanomedicines and biologicals. Mol Immunol.

[14] Szebeni J, Storm G, Ljubimova JY, Mariana Castells M, Phillips EJ, Turjeman K, Barenholz Y, Marina A. Dobrovolskaia MA, Crommelin DJA. Applying lessons learned from nanomedicines to understand rare hypersensitivity reactions to mRNA-based SARS-coV2 vaccines. Nat Nanotechnol. 2022. 10.1038/s41565-022-01071-x 10.1038/s41565-022-01071-x 35393599

[15] Szebeni J, Fontana JL, Wassef NM, Mongan PD, Morse DS, Dobbins DE, Stahl GL, Bunger R, Alving CR (1999). Hemodynamic changes induced by liposomes and liposome-encapsulated hemoglobin in pigs: a model for pseudoallergic cardiopulmonary reactions to liposomes. Role of complement and inhibition by soluble CR1 and anti-C5a antibody. Circulation.

[16] Szebeni J, Bedocs P, Csukas D, Rosivall L, Bunger R, Urbanics R (2012). A porcine model of complement-mediated infusion reactions to drug carrier nanosystems and other medicines. Adv Drug Deliv Rev.

[17] Urbanics R, Szebeni J (2015). Lessons learned from the porcine CARPA model: constant and variable responses to different nanomedicines and administration protocols. Eur J Nanomedicine.

[18] Szebeni J, Bawa R. Human clinical relevance of the porcine model of pseudoallergic infusion reactions. Biomedicines 2020;8(4):82. 10.3390/biomedicines8040082 10.3390/biomedicines8040082 PMC7235862 32276476

[19] Prescribing information, COMIRNATY® (COVID-19 Vaccine, mRNA) suspension for injection, for intramuscular use. https://www.fda.gov/media/151707/download, 2021. Accessed 19 Dec 2021

[20] Szebeni J, Baranyi L, Savay S, Bodo M, Milosevits J, Alving CR, Bunger R (2006). Complement activation-related cardiac anaphylaxis in pigs: role of C5a anaphylatoxin and adenosine in liposome-induced abnormalities in ECG and heart function. Am J Physiol Heart Circ Physiol.

[21] Kozma GT, Meszaros T, Vashegyi I, Fulop T, Orfi E, Dezsi L, Rosivall L, Bavli Y, Urbanics R, Mollnes TE, Barenholz Y, Szebeni J (2019). Pseudo-anaphylaxis to polyethylene glycol (PEG)-coated liposomes: roles of anti-PEG IgM and complement activation in a porcine model of human infusion reactions. ACS Nano.

[22] Jansen JH, Hogasen K, Mollnes TE (1993). Extensive complement activation in hereditary porcine membranoproliferative glomerulonephritis type II (porcine dense deposit disease). Am J Pathol.

[23] Zhou W, Pool V, Iskander JK, English-Bullard R, Ball R, Wise RP, Haber P, Pless RP, Mootrey G, Ellenberg SS, Braun MM, Chen RT (2003). Surveillance for safety after immunization: vaccine adverse event reporting system (VAERS)–United States, 1991–2001. MMWR Surveill Summ.

[24] Shimabukuro TT, Nguyen M, Martin D, DeStefano F (2015). Safety monitoring in the vaccine adverse event reporting system (VAERS). Vaccine.

[25] Vaccine adverse effect system. 2021. https://vaers.hhs.gov/. Accessed 19 Dec 2021

[26] Austin T, Sun S, Lim YS, Nguyen D, Lea N, Tapuria A, Kalra D (2015). An electronic healthcare record server implemented in PostgreSQL. J Healthc Eng.

[27] Doxil. DOXIL® (doxorubicin hydrochloride liposome injection) 2021.

[28] Szebeni J, Baranyi L, Savay S, Bodo M, Morse DS, Basta M, Stahl GL, Bunger R, Alving CR (2000). Liposome-induced pulmonary hypertension: properties and mechanism of a complement-mediated pseudoallergic reaction. Am J Physiol Heart Circ Physiol.

[29] ALZA Pharmaceuticals I. Doxil package insert. Mountain View, CA, USA. 2000.

[30] Barenholz Y (2012). Doxil(R)–the first FDA-approved nano-drug: lessons learned. J Control Release.

[31] Fülöp TG, Metselaar JM, Gert Storm G, Szebeni J (2017). The role of thromboxane A2 in complement activation-related pseudoallergy. Eur J Nanomed.

[32] Szebeni J, Baranyi B, Savay S, Lutz LU, Jelezarova E, Bunger R, Alving CR (2000). The role of complement activation in hypersensitivity to pegylated liposomal doxorubicin (Doxil®). J Liposome Res.

[33] Neugut AI, Ghatak AT, Miller RL (2001). Anaphylaxis in the United States: an investigation into its epidemiology. Arch Intern Med.

[34] Castells MC, Phillips EJ (2021). Maintaining safety with SARS-CoV-2 vaccines. N Engl J Med.

[35] Rama TA, Moreira A, Castells M (2021). mRNA COVID-19 vaccine is well tolerated in patients with cutaneous and systemic mastocytosis with mast cell activation symptoms and anaphylaxis. J Allergy Clin Immunol.

[36] Herve C, Laupeze B, Del Giudice G, Didierlaurent AM, Tavares Da Silva F (2019). The how's and what's of vaccine reactogenicity. NPJ Vaccines.

[37] CDC. Clinical care consideraitons for COVID-2021. https://www.cdc.gov/vaccines/covid-19/clinical-considerations/index.html. Accessed 19 Dec 2021

[38] (NIAID) NIoAaID. COVID19 SARS vaccinations: systemic allergic reactions to SARS-CoV-2 vaccinations. (ID Number: DAIT COVID-19-004). https://www.niaid.nih.gov/clinical-trials/systemic-allergic-reactions-sars-cov-2-vaccination. Accessed 19 Dec 2021

[39] Blumenthal KG, Robinson LB, Camargo CA, Shenoy ES, Banerji A, Landman AB, Wickner P (2021). Acute allergic reactions to mRNA COVID-19 vaccines. JAMA.

[40] Kozma GT, Shimizu T, Ishida T, Szebeni J (2020). Anti-PEG antibodies: properties, formation, testing and role in adverse immune reactions to PEGylated nano-biopharmaceuticals. Adv Drug Deliv Rev.

[41] Banerji A, Wickner PG, Saff R, Stone CA, Jr., Robinson LB, Long AA, Wolfson AR, Williams P, Khan DA, Phillips E, Blumenthal KG. mRNA vaccines to prevent COVID-19 disease and reported allergic reactions: current evidence and suggested approach. J Allergy Clin Immunol Pract. 2020. 10.1016/j.jaip.2020.12.047 PMC7948517 33388478

[42] Pitlick MM, Sitek AN, Kinate SA, Joshi AY, Park MA (2021). Polyethylene glycol and polysorbate skin testing in the evaluation of Coronavirus disease 2019 vaccine reactions: early report. Ann Allergy Asthma Immunol.

[43] Zhou ZH, Stone CA, Jakubovic B, Phillips EJ, Sussman G, Park J, Hoang U, Kirshner SL, Levin R, Kozlowski S (2021). Anti-PEG IgE in anaphylaxis associated with polyethylene glycol. J Allergy Clin Immunol Pract.

[44] Krantz MS, Kwah JH, Stone CA, Jr., Phillips EJ, Ortega G, Banerji A, Blumenthal KG. Safety evaluation of the second dose of messenger RNA COVID-19 vaccines in patients with immediate reactions to the first dose. JAMA Intern Med. 2021. 10.1001/jamainternmed.2021.3779 PMC8314170 34309623

[45] Szebeni J, Bedocs P, Urbanics R, Bunger R, Rosivall L, Toth M, Barenholz Y (2012). Prevention of infusion reactions to PEGylated liposomal doxorubicin via tachyphylaxis induction by placebo vesicles: a porcine model. J Control Release.

[46] Vrieze Jd. Suspicions grow that nanoparticles in Pfizer’s COVID-19 vaccine trigger rare allergic reactions. https://www.science.org/content/article/suspicions-grow-nanoparticles-pfizer-s-covid-19-vaccine-trigger-rare-allergic-reactions. Accessed 19 Dec 2021

[47] Szebeni J (2001). Complement activation-related pseudoallergy caused by liposomes, micellar carriers of intravenous drugs and radiocontrast agents. Crit Rev Ther Drug Carr Syst.

[48] Szebeni J (2005). Complement activation-related pseudoallergy: a new class of drug-induced acute immune toxicity. Toxicology.

[49] Moghimi SM (2021). Allergic Reactions and anaphylaxis to LNP-based COVID-19 vaccines. Mol Ther J Am Soc Gene Ther.

[50] Chanan-Khan A, Szebeni J, Savay S, Liebes L, Rafique NM, Alving CR, Muggia FM (2003). Complement activation following first exposure to pegylated liposomal doxorubicin (Doxil): possible role in hypersensitivity reactions. Ann Oncol.

[51] Fernandez HN, Hugli TE (1976). Partial characterization of human C5a anaphylatoxin. I. Chemical description of the carbohydrate and polypeptide prtions of human C5a. J Immunol.

[52] Gorski JP, Hugli TE, Muller-Eberhard HJ (1979). C4a: the third anaphylatoxin of the human complement system. Proc Natl Acad Sci U S A.

[53] Gold MS, MacDonald NE, McMurtry CM, Balakrishnan MR, Heininger U, Menning L, Benes O, Pless R, Zuber PLF (2020). Immunization stress-related response - redefining immunization anxiety-related reaction as an adverse event following immunization. Vaccine.

[54] Kelso JM (2021). Misdiagnosis of systemic allergic reactions to mRNA COVID-19 vaccines. Ann Allergy Asthma Immunol.

[55] Kelso JM. Covid-19: allergic-reactions-to-sars-cov-2-vaccines. https://www.uptodate.com/contents/covid-19-allergic-reactions-to-sars-cov-2-vaccines. Accessed 19 Dec 2021

[56] Hause AM, Gee J, Johnson T, Jazwa A, Marquez P, Miller E, Su J, Shimabukuro TT, Shay DK (2021). Anxiety-related adverse event clusters after Janssen COVID-19 vaccination - five U.S. mass vaccination sites, April 2021. MMWR Morb Mortal Wkly Rep.

[57] Hoffman Y, Palgi Y, Goodwin R, Ben-Ezra M, Greenblatt-Kimron L. A storm in a teacup: older adults' low prevalence of COVID-19 vaccine side-effects and their link with vaccination anxiety. Int Psychogeriatr. 2021:1–3. 10.1017/S1041610221001071 34488916

[58] Hugli TE, Stimler NP, Gerard C, Moon KE (1981). Possible role of serum anaphylatoxins in hypersensitivity reactions. Int Arch Allergy Appl Immunol.

[59] Reber LL, Hernandez JD, Galli SJ (2017). The pathophysiology of anaphylaxis. J Allergy Clin Immunol.

[60] Lepow IH, Willms-Kretschmer K, Patrick RA, Rosen FS (1970). Gross and ultrastructural observations on lesions produced by intradermal injection of human C3a in man. Am J Pathol.

[61] Wuepper KD, Bokisch VA, Muller-Eberhard HJ, Stoughton RB (1972). Cutaneous responses to human C 3 anaphylatoxin in man. Clin Exp Immunol.

[62] Yancey KB, Hammer CH, Harvath L, Renfer L, Frank MM, Lawley TJ (1985). Studies of human C5a as a mediator of inflammation in normal human skin. J Clin Invest.

[63] Szebeni J, Stafil G, Fontana J, Dobbins D, Mongan P, Wassef N, Morse D, Bunger R, Alving C. Liposome-induced acute cardiopulmonary distress in swine is mediated by complement. Circulation. 1998;98(17):133–133.

[64] Szebeni J (2018). Mechanism of nanoparticle-induced hypersensitivity in pigs: complement or not complement?. Drug Discov Today.

[65] Szebeni J, Bedőcs P, Dézsi L, Urbanics R (2018). A porcine model of complement activation-related pseudoallergy to nano-pharmaceuticals: pros and cons of translation to a preclinical safety test. Prec Nanomed.

[66] Alving CR (1984). Natural antibodies against phospholipids and liposomes in humans. Biochem Soc Trans.

[67] Swartz GM, Gentry MK, Amende LM, Blanchette-Mackie EJ, Alving CR (1988). Antibodies to cholesterol. Proc Natl Acad Sci USA.

[68] Jiang H, Cooper B, Robey FA, Gewurz H (1992). DNA binds and activates complement via residues 14–26 of the human C1q A chain. J Biol Chem.

[69] Chonn A, Cullis PR, Devine DV (1991). The role of surface charge in the activation of the classical and alternative pathways of complement by liposomes. J Immunol.

[70] Plank C, Mechtler K, Szoka FC, Wagner E (1996). Activation of the complement system by synthetic DNA complexes: a potential barrier for intravenous gene delivery. Hum Gene Ther.

[71] Ali YM, Ferrari M, Lynch NJ, Yaseen S, Dudler T, Gragerov S, Demopulos G, Heeney JL, Schwaeble WJ (2021). Lectin pathway mediates complement activation by SARS-CoV-2 proteins. Front Immunol.

[72] Selmin F, Musazzi UM, Franze S, Scarpa E, Rizzello L, Procacci P, Minghetti P (2021). Pre-drawn syringes of Comirnaty for an efficient COVID-19 mass vaccination: demonstration of stability. Pharmaceutics.

[73] Nogueira S, Schlegel A, Maxeiner K, Weber B, Barz M, Schroer MA, Blanchet CE, Svergun DI, Ramishetti S, Peer D, Langguth P, Sahin U, Haas H (2020). Polysarcosine-Functionalized lipid nanoparticles for therapeutic mRNA delivery. ACS Appl Nano Mater.

[74] Sedic M, Senn JJ, Lynn A, Laska M, Smith M, Platz SJ, Bolen J, Hoge S, Bulychev A, Jacquinet E, Bartlett V, Smith PF (2018). Safety evaluation of lipid nanoparticle-formulated modified mRNA in the Sprague-Dawley rat and cynomolgus monkey. Vet Pathol.

[75] McMahon DE, Amerson E, Rosenbach M, Lipoff JB, Moustafa D, Tyagi A, Desai SR, French LE, Lim HW, Thiers BH, Hruza GJ, Blumenthal KG, Fox LP, Freeman EE (2021). Cutaneous reactions reported after Moderna and Pfizer COVID-19 vaccination: a registry-based study of 414 cases. J Am Acad Dermatol.

[76] Blumenthal KG, Freeman EE, Saff RR, Robinson LB, Wolfson AR, Foreman RK, Hashimoto D, Banerji A, Li L, Anvari S, Shenoy ES (2021). Delayed large local reactions to mRNA-1273 vaccine against SARS-CoV-2. N Engl J Med.

[77] Kolev M, Le Friec G, Kemper C (2014). Complement–tapping into new sites and effector systems. Nat Rev Immunol.

[78] Ozbakir B, Crielaard BJ, Metselaar JM, Storm G, Lammers T (2014). Liposomal corticosteroids for the treatment of inflammatory disorders and cancer. J Control Release.

[79] Wong AD, Ye M, Ulmschneider MB, Searson PC (2015). Quantitative analysis of the enhanced permeation and retention (EPR) effect. PLoS One.

[80] Watanabe A, Tanaka H, Sakurai Y, Tange K, Nakai Y, Ohkawara T, Takeda H, Harashima H, Akita H (2016). Effect of particle size on their accumulation in an inflammatory lesion in a dextran sulfate sodium (DSS)-induced colitis model. Int J Pharm.

[81] Borg T, Gerdin B, Hallgren R, Warolin O, Modig J (1984). Complement activation and its relationship to adult respiratory distress syndrome. An experimental study in pigs. Acta Anaesthesiol Scand.

[82] Marceau F, Lundberg C, Hugli TE (1987). Effects of anaphylatoxins on circulation. Immunopharmacol.

[83] Chanan-Khan A, Szebeni J, Leibes L, Rafique M, Savay S, Alving CR, Muggia FM. Complement activation by pegylated liposomal doxorubicin (DoxilR) in cancer patients: association with hypersensitivity reactions. J Control Drug Rel. 2001;abstract, in press.

[84] Szebeni J, Simberg D, Gonzalez-Fernandez A, Barenholz Y, Dobrovolskaia MA (2018). Roadmap and strategy for overcoming infusion reactions to nanomedicines. Nat Nanotechnol.

[85] Meszaros T, Kozma GT, Shimizu T, Miyahara K, Turjeman K, Ishida T, Barenholz Y, Urbanics R, Szebeni J (2018). Involvement of complement activation in the pulmonary vasoactivity of polystyrene nanoparticles in pigs: unique surface properties underlying alternative pathway activation and instant opsonization. Int J Nanomedicine.

[86] Elsabahy M, Li A, Zhang F, Sultan D, Liu Y, Wooley KL (2013). Differential immunotoxicities of poly(ethylene glycol)- vs. poly(carboxybetaine)-coated nanoparticles. J Control Release.

[87] Elsabahy M, Wooley KL (2013). Cytokines as biomarkers of nanoparticle immunotoxicity. Chem Soc Rev.

[88] Chaplin H (2005). Review: the burgeoning history of the complement system 1888–2005. Immunohematology.

[89] Bavli Y, Winkler I, Chen BM, Roffler S, Cohen R, Szebeni J, Barenholz Y (2019). Doxebo (doxorubicin-free Doxil-like liposomes) is safe to use as a pre-treatment to prevent infusion reactions to PEGylated nanodrugs. J Control Release.

[90] Kasperovic P. Dosages and methods for delivering lipid formulated nucleic acid molecules. US patent applicationsd #20160122759. 2016.

[91] Fulop T, Kozma GT, Vashegyi I, Meszaros T, Rosivall L, Urbanics R, Storm G, Metselaar JM, Szebeni J (2019). Liposome-induced hypersensitivity reactions: risk reduction by design of safe infusion protocols in pigs. J Control Release.

[92] Klimek L, Novak N, Cabanillas B, Jutel M, Bousquet J, Akdis CA. Allergenic components of the mRNA-1273 vaccine for COVID-19: possible involvement of polyethylene glycol and IgG-mediated complement activation. Allergy. 2021. 10.1111/all.14794 PMC8013891 33657648

[93] Murphy KR, Patel NC, Ein D, Hudelson M, Kodoth S, Marshall GD, Parikh P, Blaiss MS (2021). Insights from American College of Allergy, Asthma, and Immunology COVID-19 vaccine task force: allergic reactions to mRNA SARS-CoV-2 vaccines. Ann Allergy Asthma Immunol.

[94] Kricorian K, Civen R, Equils O. COVID-19 vaccine hesitancy: misinformation and perceptions of vaccine safety. Hum Vaccin Immunother. 2021:1–8. 10.1080/21645515.2021.1950504 PMC8920251 34325612

[95] Ara Y, Saito T, Takagi T, Hagiwara E, Miyagi Y, Sugiyama M, Kawamoto S, Ishii N, Yoshida T, Hanashi D, Koshino T, Okada H, Okuda K (2001). Zymosan enhances the immune response to DNA vaccine for human immunodeficiency virus type-1 through the activation of complement system. Immunology.

[96] Stager S, Alexander J, Kirby AC, Botto M, Rooijen NV, Smith DF, Brombacher F, Kaye PM (2003). Natural antibodies and complement are endogenous adjuvants for vaccine-induced CD8+ T-cell responses. Nat Med.

[97] Mattsson J, Yrlid U, Stensson A, Schon K, Karlsson MC, Ravetch JV, Lycke NY (2011). Complement activation and complement receptors on follicular dendritic cells are critical for the function of a targeted adjuvant. J Immunol.

[98] Hung CY, Hurtgen BJ, Bellecourt M, Sanderson SD, Morgan EL, Cole GT (2012). An agonist of human complement fragment C5a enhances vaccine immunity against Coccidioides infection. Vaccine.

[99] Dempsey PW, Allison ME, Akkaraju S, Goodnow CC, Fearon DT (1996). C3d of complement as a molecular adjuvant: bridging innate and acquired immunity. Science.

[100] Morgan BP, Marchbank KJ, Longhi MP, Harris CL, Gallimore AM (2005). Complement: central to innate immunity and bridging to adaptive responses. Immunol Lett.

